# Automated Chronic Obstructive Pulmonary Disease Phenotyping and Control Assessment in Primary Care: Retrospective Multicenter Study Using the Seleida Model

**DOI:** 10.2196/74932

**Published:** 2025-10-13

**Authors:** José David Maya Viejo, Fernando M Navarro Ros

**Affiliations:** 1 Centro de Salud de Camas, Santa María de Gracia 54Seville, 41900, Spain, 34 955 01 94 60; 2 Centro de Salud Plaza Segovia, Valencia, Spain

**Keywords:** COPD, phenotyping, predictive model, poor control, primary care, exacerbations, risk estimation, electronic health record, health care utilization, precision medicine, chronic obstructive pulmonary disease

## Abstract

**Background:**

Chronic obstructive pulmonary disease (COPD) remains a leading global health burden. In primary care, the inconsistent availability of spirometry and symptom scores limits the detection of patients with poor disease control. There is a pressing need for scalable, data-driven tools that leverage routinely collected clinical information to support timely, equitable, and guideline-concordant interventions.

**Objective:**

This study aims to validate the performance of Seleida—a fully automated, deterministic, and bijective model for COPD control assessment and phenotyping—using real-world primary care data and to evaluate its feasibility for integration into electronic health record (EHR)–based informatics systems.

**Methods:**

Seleida estimates the probability of poor control (*Pr*) using two objective EHR variables: (1) annual dispensations of short-acting bronchodilators—specifically short-acting β2-agonists (SABA), short-acting muscarinic antagonists (SAMA), or both, and (2) number of dispensed antibiotic courses for bronchitis or COPD exacerbations. Its bijective structure supports both forward risk estimation and reverse phenotype inference. In a retrospective cohort of 106 patients, agreement was assessed between 2 phenotyping systems (a 126-combination model and a streamlined 21-combination version) and with clinician-assigned classifications. Due to sample size limitations, a provisional risk threshold of *Pr*>.50 was adopted for internal stratification.

**Results:**

Seleida showed perfect agreement between phenotyping systems (Cohen κ=1.00; *P*<.001) and substantial concordance with clinician-assigned profiles (Cohen κ=0.70; *P*<.001). The model operates transparently, without machine learning, and can be embedded into EHR platforms or applied manually using a visual framework. It enables individualized risk estimation, phenotype-driven treatment planning, and population-level case identification—particularly in settings with limited access to traditional diagnostic tools.

**Conclusions:**

Seleida provides a reproducible and interpretable framework for COPD control monitoring using high-frequency prescribing data. Its transparent logic, low data burden, and interoperability enable integration across diverse digital infrastructures, including resource-limited settings. By supporting both individualized care and population-level risk stratification, Seleida bridges predictive analytics with real-world clinical decision-making. Ongoing multicenter validation will determine its generalizability, clinical impact, and cost-effectiveness at scale.

## Introduction

Chronic obstructive pulmonary disease (COPD) remains a major global health challenge, with more than 3.2 million deaths annually and more than 390 million people affected worldwide [1,2]. In Europe, direct health care costs exceed $56.5 billion per year (the cost that was originally reported in euros [€] was converted to US dollars using the exchange rate of €1=$1.18 as of September 15, 2025, based on the European Central Bank reference rates) [3], with the burden concentrated in primary care, where early interventions are most feasible [4].

Yet in real-world settings, early identification of patients at risk of exacerbation or poor control remains limited. Nearly 50% of individuals with COPD experience at least 1 moderate to severe exacerbation annually, often without prior clinical detection [5-8]. These events are not only indicators of disease instability but also independent risk factors for cardiovascular morbidity and mortality. Large-scale studies have shown sharply increased risks of death and cardiovascular events—such as myocardial infarction, heart failure, stroke, and arrhythmias—within 30 days of an exacerbation, with elevated risk persisting for up to a year [9-12].

The underlying pathophysiology—driven by systemic inflammation, hypoxia, and prothrombotic states—may explain this sustained vulnerability [10,11]. The effect is cumulative: each subsequent exacerbation further increases cardiopulmonary risk [9]. These episodes also drive disproportionate health care use and cost, particularly via emergency and hospital care [11,13].

In this context, it is essential to develop tools that not only predict a patient’s level of COPD control but also accurately phenotype them, since therapeutic optimization depends heavily on phenotype-specific characteristics. Personalized inhaled treatment, guided by phenotype, has the potential to reduce exacerbations and mitigate their associated systemic and economic consequences.

While current frameworks—such as Global Initiative for Chronic Obstructive Lung Disease (GOLD) 2025 and GesEPOC 2021—guide treatment decisions, they rely on clinical data often unavailable in primary care, including spirometry and symptom scales [14-17]. Moreover, they underutilize objective, high-frequency prescribing data—such as rescue bronchodilator or antibiotic use—despite their predictive value for clinical instability [12].

To address this gap, we developed Seleida: a fully automated classification system that is deterministic, meaning it produces the same output for any given input without randomness, and bijective, meaning each patient profile corresponds to a unique, reversible clinical category. The model is based solely on 2 universally available variables in structured electronic health records (EHRs): the annual number of short-acting β2-agonists (SABA) and short-acting muscarinic antagonists (SAMA) dispensations, or both, and the annual number of antibiotic dispensations for respiratory events, specifically bronchitis and COPD exacerbations [18-20]. These variables serve as robust proxies for symptom burden and exacerbation frequency, facilitating seamless integration into routine clinical workflows [21-23].

By intentionally excluding low-access metrics (eg, forced expiratory volume in the first second of expiration, patient-reported outcomes), Seleida prioritizes scalability and feasibility without sacrificing predictive accuracy. Prior work suggests minimal added value from incorporating such variables into risk models [18,20].

This study aimed to validate the Seleida model using real-world data from 2 Spanish primary care centers. We hypothesized that a deterministic, fully automated system based solely on prescribing data—specifically, annual SABA or SAMA and antibiotic dispensations—could reliably stratify patients with COPD by control status and phenotype, without relying on spirometry or symptom scales. To this end, the study pursued 3 interrelated goals: validating the model’s bijective structure, comparing model-derived phenotypes against the 2025 GOLD ABE classification and between the 126- and 21-combination configurations, and evaluating Seleida’s clinical use for early risk identification and digital deployment in routine care.

## Methods

### Study Design, Setting, and Population

This retrospective, multicenter pilot cohort validation study was nested within the Seleida project—an initiative to develop scalable models, based on EHRs, for identifying poor control in chronic respiratory diseases. The Seleida model had been previously developed and internally validated using two consistently recorded variables in structured EHRs: (1) annual dispensations of rescue inhalers (SABA, SAMA, or both) and (2) respiratory antibiotics [24,25]. These variables were chosen for their clinical relevance, high traceability, and interoperability with primary care informatics systems [21-23].

For this validation study, anonymized patient-level EHR data were extracted from 2 urban primary care centers in Seville and Valencia, both integrated within the Spanish National Health System [21,26]. These centers serve demographically diverse populations and represent real-world contexts for Seleida deployment.

From a reference population of 82,631 adults, a stratified random sample of 110 individuals aged 40‐80 years was drawn. Selection was based on the presence of a COPD diagnosis (*International Classification of Diseases, Tenth Revision* [*ICD-10*]), on compatible pharmacological treatment for at least 3 months per year over the previous 2 years, or on both criteria [6,8]. Predefined exclusion criteria were active malignancy, palliative care, systemic corticosteroids or biologics for nonrespiratory indications, pregnancy, severe disability, enrollment in clinical trials, asthma-COPD overlap syndrome, and absence of recorded pharmacological activity during the 12-month observation period.

All dispensing records were cross-validated with linked pharmacy databases to minimize misclassification bias from unfilled or partially filled prescriptions [27,28]. Variables with more than 50% missing data were excluded, following standard epidemiological guidance [28,29]. Operational definitions, measurement units, and coding conventions are described in section S2 in Multimedia Appendix 1.

Four patients (4/6, 3.6%) met exclusion criteria, leaving a final analytical cohort of 106 patients (60/106, 56.6%) from Seville and from Valencia (46/106, 43.4%), with a mean age of 68.8 (SD 8.2) years; most were male (77/106, 72.6%).

To ensure data integrity, automated quality checks were performed to detect missing fields and inconsistencies. Data processing and storage followed international health informatics standards [27,30]. The methodological infrastructure was designed to support interoperability with HL7 FHIR (Health Level Seven Fast Healthcare Interoperability Resources; see section S7 in Multimedia Appendix 1), enabling future integration into clinical decision support tools [21,22,31-33].

### Objective and Methodology

The primary aim of this study was to validate the predictive performance and mathematical structure of the Seleida model—a deterministic and bijective algorithm designed for real-time COPD control assessment—using structured, real-world EHR data from primary care. The secondary aim was to evaluate its feasibility for integration into digital health infrastructures and its ability to support automated phenotyping in routine clinical workflows.

To address the primary aim, the study pursued 3 interrelated objectives:

1. To validate Seleida’s bijective structure, which ensures a one-to-one correspondence between clinical inputs and predicted risk estimates. It guarantees that each unique combination of rescue inhaler use and exacerbation frequency produces a distinct, reproducible phenotypic classification [20,34].

2. To compare Seleida’s model–derived phenotypes with established clinical classifications based on the 2025 GOLD guidelines. This included:

- Comparing the 126-combination high-resolution phenotyping system to the guideline-defined 2025 GOLD ABE classification [27,35],
- Comparing the 21-combination streamlined system to the 2025 GOLD ABE classification [4], and
- Assessing the agreement between the 126- and 21-combination structures.

Both systems support two phenotyping methods: (1) basic phenotyping (closely aligned with the ABE classification in GOLD 2025) and (2) expanded phenotyping (combines the ABE classification with stratification based on SABA use levels—low or high: L or H), offering a nuanced characterization aligned with Seleida’s risk profiles.

3. To assess the model’s clinical use for early risk detection and phenotype-driven decision support. The goal is to enable proactive, data-driven interventions for poorly controlled patients in EHR-integrated environments [20,36].

By combining mathematical determinism with real-world clinical traceability, Seleida offers a scalable and interpretable alternative to conventional risk classification systems. Its design supports automated control monitoring, granular patient segmentation, and guideline-concordant decision-making in both high- and low-resource care settings [34,37,38].

### Outcome Definition and Model Application

The operational definition of poor control in COPD was adapted to align with Seleida’s data-driven thresholds: ≥1 moderate exacerbation (requiring antibiotics or systemic corticosteroids without hospitalization), ≥1 severe exacerbations (requiring hospitalization or emergency room visit), or annual use of at least 3 SABA or SAMA canisters or both [14,17,25]. This definition enables continuous risk stratification and phenotype inference without the need for spirometry or symptom scales, improving scalability in digital health environments. By these criteria, 55.7% (59/106) of the cohort was classified as poorly controlled. Control status was evenly distributed across sites (*χ*²_1_=1.055; *P*=.304), and baseline demographic and clinical characteristics were compared for balance [39-41].

### Model Development, Validation, and Performance Evaluation

The analytical cohort comprised 106 patients, selected pragmatically based on feasibility and validated against formal criteria for clinical prediction modeling. With a binary outcome (poor vs good control) and a 55.7% (59/106) prevalence of poor control, the training subset (85/106, 80% of the cohort) included approximately 47 events. This yielded an events-per-variable ratio of 23.5 for the 2 selected predictors—annual rescue inhaler canisters and respiratory antibiotic courses dispensed—well above the recommended ≥10 events-per-variable threshold for stable logistic regression estimates [29,41,42].

Additional clinical and demographic variables—age, sex, geographic origin, exacerbation history, daily inhalation frequency, and emergency visits—were collected for descriptive purposes and evaluated for potential inclusion. An exploratory multivariable logistic regression including age group, sex, SABA or SAMA use, or both, and antibiotic prescriptions confirmed the independent predictive strength of the 2 primary variables (*P*=.001 and *P*<.001, respectively). In contrast, age and sex were not statistically significant (*P*=.65 and *P*=.65), and their inclusion caused numerical instability due to sparse subgroup representation (eg, patients aged 80 years without exacerbations or females with high SABA or SAMA use or both). This led to quasi-complete separation and convergence failures, manifested as inflated standard errors, infinite odds ratios [Exp(β) >10⁸], and singularities in the Hessian matrix—classic indicators of model overspecification in small samples. Full diagnostics and regression results are detailed in section S5 in Multimedia Appendix 1. Following best practices for parsimonious and deployable models [4,6], age and sex were excluded from the final Seleida model to reduce overfitting, preserve statistical stability, and maintain generalizability in small or imbalanced datasets.

The final Seleida model, including only the 2 primary predictors, achieved strong predictive performance in the initial validation: area under the receiver operating characteristic curve of 0.978, sensitivity of 92.86%, specificity of 87.50%, κ coefficient of 0.80, and positive likelihood ratio (LR+) of 7.43. Bootstrap resampling (1000 iterations) confirmed metric stability, supporting both clinical use and statistical robustness [24,25].

Internal validity was also assessed with bootstrap resampling (1000 iterations), following Steyerberg’s framework for early-phase models [25,29]. The α level was set at .05, and the minimum detectable effect size was estimated from the observed event distribution and prior literature [24,25]. In addition, 10,000 Monte Carlo simulations using real-world covariate distributions indicated a model-level statistical power (1–β) of 92.4% (95% CI 91.85‐92.87), supporting high reliability for detecting clinically relevant effects in this sample and predictor configuration. Bootstrap-derived CIs quantified uncertainty in coefficient estimates and performance metrics.

All analyses were conducted using R (version 4.3.4.2; R Core Team), following established best practices for early-phase predictive modeling. Model development used logistic regression with Least Absolute Shrinkage and Selection Operator (LASSO) regularization to enhance parsimony and reduce overfitting in a small to moderate sample context (section S4 in Multimedia Appendix 1) [33]. Predictors were modeled without scaling to preserve clinical interpretability.

Building on this validated model, this study evaluates 2 deterministic phenotyping frameworks derived from its final equation: a 126-combination system and a simplified 21-combination version. Both were assessed using classification-based performance metrics—sensitivity, specificity, predictive values, LRs, and Cohen κ—against real-world clinical phenotypes. Internal concordance between systems was evaluated using κ statistics. Additionally, the 126-combination system was also benchmarked against the 2025 GOLD ABE classification to assess alignment with guideline-based stratification [22,31,43,44].

Furthermore, the simplified phenotyping system was evaluated for computational efficiency and its potential integration into HL7 FHIR–compliant health information systems (section S7 in Multimedia Appendix 1) [22,31,32]. All procedures followed TRIPOD guidelines and contemporary standards for internal validation of clinical prediction models [45,46].

### Reverse Model Definition

A defining feature of the Seleida model is its deterministic and bijective architecture, establishing a one-to-one correspondence between 2 routinely collected clinical variables and the predicted probability of poor COPD control (sections S1 and S2 in Multimedia Appendix 1). Specifically:

- ***a*** represents the annual number of SABA or SAMA canisters or both dispensed per patient, serving as a proxy for symptom burden and short-acting bronchodilator dependence [18,47].
- ***b*** denotes the annual number of antibiotic dispensations for bronchitis or COPD exacerbations, reflecting exacerbation frequency and infection-driven instability [48,49].

Both variables were chosen for their objectivity, universal availability in structured EHRs, and strong predictive value in prior studies [25,50]. The predicted probability of poor control (*Pr*) is calculated with a logistic regression model:


$$logit(Pr)=\alpha +\beta_{1}\cdot a+\beta_{2}\cdot b,$$

where α is the intercept, and β₁ and β₂ are positive coefficients estimated using LASSO regularization, ensuring parsimony and coefficient stability (section S4 in Multimedia Appendix 1).

This structure supports two complementary functions: (1) forward inference: estimating *Pr* from any (*a, b*) combination, and (2) reverse inference: retrieving the (*a, b*) pairs matching a target risk level. The latter enables real-time, automated phenotyping and individualized decision-making in EHR-integrated workflows.

Although Seleida can estimate risk directly from observed data, it is explicitly designed for bidirectional operation. This means that it can both predict risk from clinical inputs and perform inverse mapping to retrieve all (*a, b*) pairs that match a given probability. This reverse functionality is critical for dynamic alert systems, phenotype-driven decision pathways, and real-time decision support tools.

This bidirectional capability has practical implications. For example, a patient with 3 annual SABA or SAMA inhalers or both (*a*=3) and 1 antibiotic course (*b*=1) maps to a unique cell (3,1) in the 126-combination system, with a predicted poor control risk of 95.53%. In the simplified 21-combination version, the same profile is classified as *a*=3 (midrange inhaler use) and *b*=1 (moderate exacerbation frequency), preserving its phenotypic identity—GOLD A1/B1 with high rescue inhaler use (H)—within a reduced but clinically actionable framework. This structured mapping supports therapeutic reassessment and integration into EHR-based alert systems, where predefined thresholds (eg, *Pr*>.50) can trigger guideline-aligned interventions.

In contrast to black box machine learning models, often criticized for limited interpretability, Seleida’s transparent structure ensures full traceability from risk estimate to actionable clinical profile. This enhances clinical confidence and facilitates deployment across diverse health care settings, including resource-constrained environments [20,34,37,38]. A detailed mathematical demonstration of these bijective properties, with exhaustive verification of all input-output mappings, appears in the following section and in section S3 in Multimedia Appendix 1.

### Mathematical Proof of Bijectivity

Bijectivity is not merely a theoretical refinement but a functional cornerstone of the Seleida model. As detailed in section S1, its formal proof guarantees three essential capabilities: (1) each predicted probability is uniquely traceable to a specific clinical phenotype, ensuring transparency and auditability; (2) every clinically relevant risk threshold corresponds to at least 1 valid input combination, providing full operational coverage; and (3) no ambiguity or discontinuity occurs in model behavior, maintaining consistency in decision support and regulatory contexts. Without this formal validation, the model’s dual interpretability and reproducibility would remain unverified.

To confirm these properties, we validated that each (*a, b*) input pair yields a unique predicted probability (*injectivity*), and that the entire risk spectrum (0<*Pr*<1) is covered by the input domain (*surjectivity*). These conditions are defined as follows:

- Injectivity: Each unique (*a, b*) pair—representing the annual number of dispensed SABA or SAMA canisters or both and respiratory antibiotics—must map to a single, distinct predicted probability of poor COPD control (*Pr*).
- Surjectivity: Every probability value within the model’s codomain must be attainable from at least 1 clinically plausible (*a, b*) combination, ensuring that there are no gaps in the risk scale.

We followed a rigorous, multistep validation process to formally demonstrate the bijective nature of the Seleida model:

- Bounding inputs: The variables—annual SABA or SAMA canisters or both (*a*) and annual antibiotic regimens (*b*)—were explicitly restricted to integers: *a*=0‐20 and *b*=0‐5 (inclusive). These ensured a finite, fully enumerable input space.
- Analytical proof of injectivity: We demonstrated that no 2 distinct input pairs (*a*₁, *b*₁) and (*a*₂, *b*₂) produce the same output probability. The logistic model’s coefficients structure confirmed unique mappings for all valid pairs.
- Computational proof of surjectivity: Using a custom Python algorithm, we calculated all 126 possible output probabilities to 14 decimal places. Every value was checked for uniqueness, confirming that the entire codomain is covered.
- Conclusion: Independent confirmation of both injectivity and surjectivity verified that Seleida is bijective, enabling forward risk estimation and reverse phenotype reconstruction with mathematical certainty.

No repeated or missing outputs were found, confirming a perfect alignment between input and output spaces. This property ensures robust reverse inference and reproducible phenotype classification across real-world COPD datasets. All source code, verification tables, and derivations are provided in section S3 in Multimedia Appendix 1 [45,51].

### Ethical Considerations

The study was approved by the ethics committee of Hospital Universitario Doctor Peset of Valencia (protocol code CEIm 132.22, approval date March 6, 2023). The project was registered in the *Portal de Ética de la Investigación Biomédica de Andalucía* through the *Sistema de Información de los Comités de Ética de la Investigación* (protocol code 1140-N-23, approval date September 12, 2023). The SEMERGEN research department gave its endorsement to the project (2023-00035, approval date June 6, 2023) [27,28]. For the purpose of this study, only patient data recorded in EHRs were collected. All data were fully anonymized in compliance with the General Data Protection Regulation (2016/679). No procedures were performed on participants and, to ensure confidentiality, each patient was assigned a unique study identifier. Informed consent was formally waived due to the retrospective design and the absence of patient contact. Due to the nature of the study, compensation to patients was not applicable.

## Results

### Expanded Phenotyping: Analysis of the 126-Combination System

**Figure 1.**
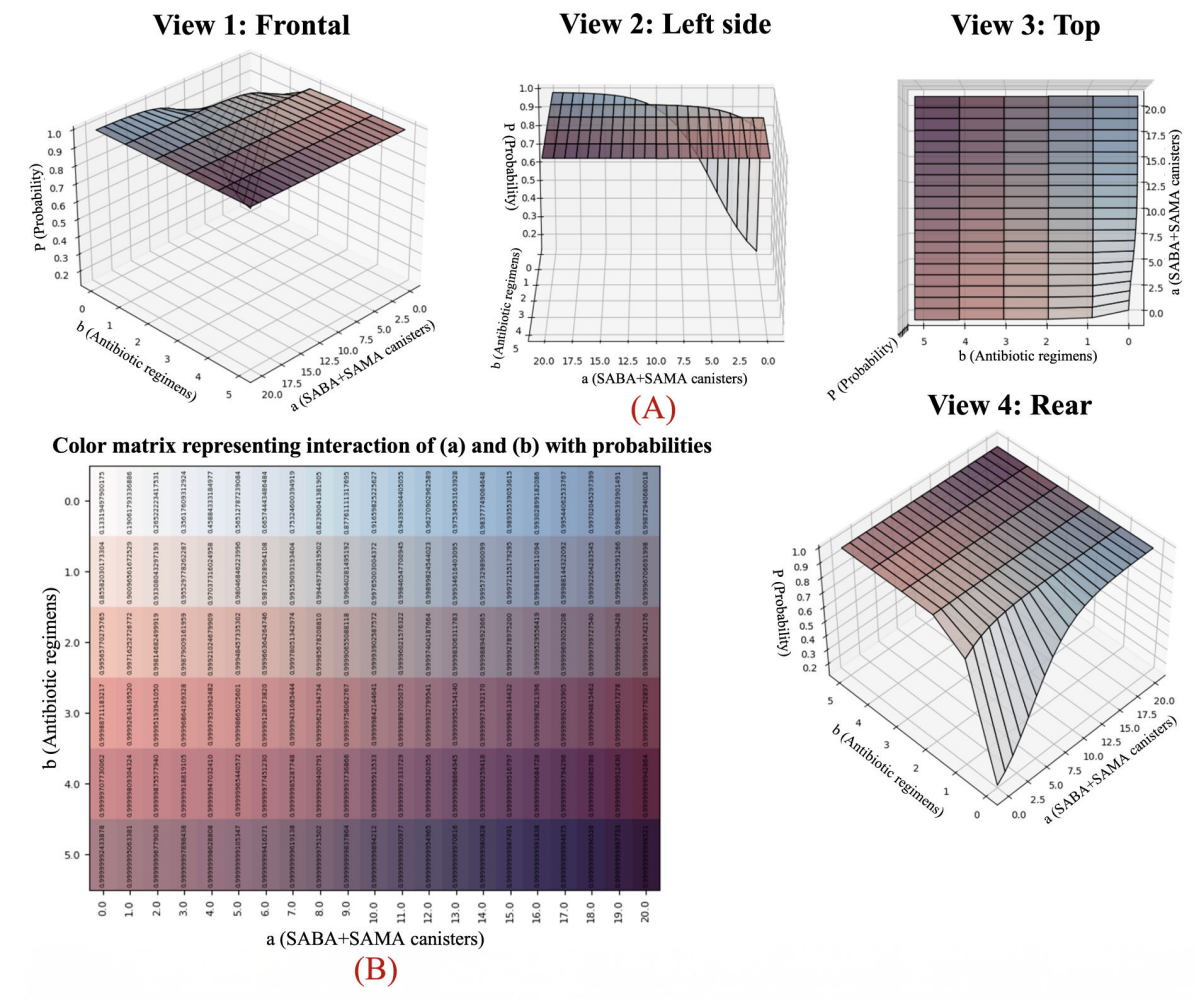
Visual mapping of the Seleida model’s 126-combination system. The 4 images in panel (A) show 3D perspectives of how annual rescue inhaler use (*a*) and antibiotic prescriptions (*b*) combine to yield the probability of poor chronic obstructive pulmonary disease control (*Pr*). Panel (B) provides a color matrix of exact *Pr* values across all combinations. Darker red indicates higher predicted risk. Clinically, higher values of *b* (≥2 antibiotics per year) are stronger drivers of poor control than *a*, highlighting infection burden as a key modifiable factor. SABA: short-acting β2-agonists; SAMA: short-acting muscarinic antagonists.

The 126-combination phenotyping framework systematically integrates 2 structured prescribing variables—annual dispensations of rescue inhalers (*a*) and respiratory antibiotic regimens (*b*)—to generate a continuous, 3D map of predicted COPD control probabilities (Figure 1A and B). Blue gradients reflect increasing use of SABA or SAMA canisters or both (*a*), red gradients indicate the frequency of antibiotic courses (*b*), and purple regions highlight zones of sharply elevated clinical risk [35]. Each unique (*a, b*) pair yields a distinct probability estimate, delineating a mathematically robust and clinically interpretable risk surface with actionable thresholds [20,34].

To guarantee bijectivity, the input domain was discretized to include integer values from 0 to 20 for *a* and from 0 to 5 for *b* (both inclusive). Any observed values exceeding these bounds are computationally capped at *a*=20 and *b*=5, preserving full injectivity and surjectivity across the model’s codomain. This bounded structure ensures reversibility and enables exhaustive phenotype enumeration while maintaining operational realism.

Model outputs demonstrate that antibiotic burden (*b*) exerts a greater marginal influence on the predicted probability of poor control than rescue bronchodilator use (*a*), particularly within intermediate strata. For example, the phenotype (*a*=3, *b*=1) is associated with substantially elevated risk, whereas combinations with *a *≥ 15 and *b *≥ 4 approach probability ceilings exceeding 99% [36,52]. These gradients reflect the compounding effects of symptom burden and exacerbation frequency, underscoring the need for integrated monitoring of both variables in routine care.

By translating complex statistical interactions into visually intuitive gradients, the 126-combination system provides clinicians with a practical, high-resolution tool for individualized COPD phenotyping—enabling phenotype-driven interventions, anticipatory care strategies, and optimized treatment planning (section S6 in Multimedia Appendix 1) [4,34,38].

### Expanded Phenotyping: Analysis of the 21-Combination System

To enhance feasibility in real-world clinical settings, the original 126-combination system was streamlined into a 21-combination framework by constraining the antibiotic regimen variable (*b*) to a range of 0‐2 and the SABA or SAMA canisters or both variable (*a*) to 0‐6. These thresholds were empirically defined to preserve the model’s bijective architecture while concentrating on clinically meaningful stratification intervals [4,20,24].

**Figure 2.**
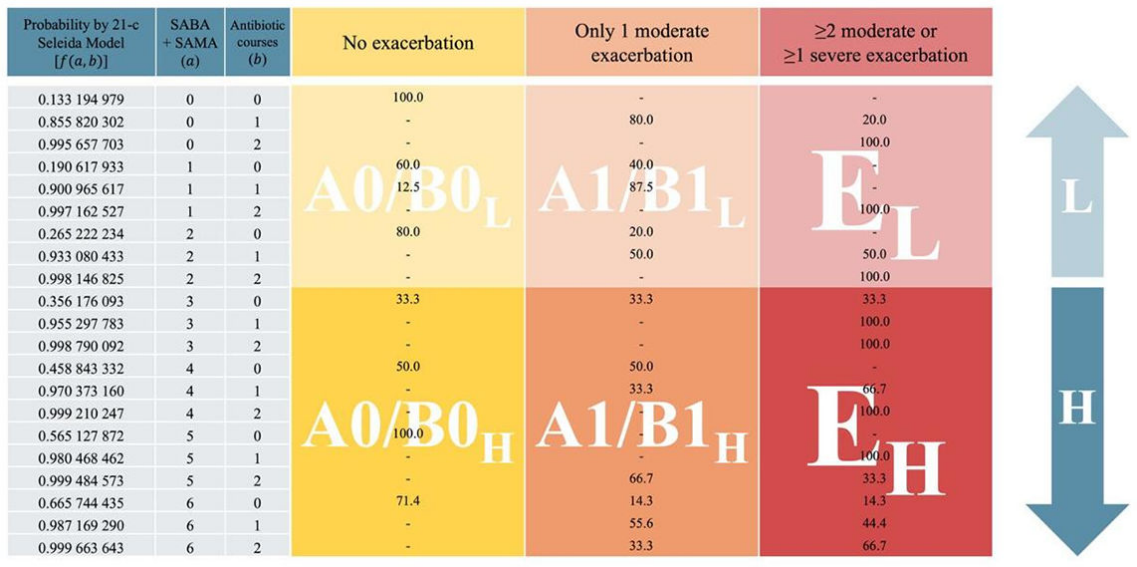
Chronic obstructive pulmonary disease phenotyping using the simplified 21-combination Seleida system. Each cell reflects the predicted risk level based on rescue inhaler use (*a*) and antibiotic courses (*b*), capped at clinically relevant thresholds (*a*≤6; *b*≤2). Color intensity indicates increasing probability of poor control. This tool enables rapid identification of high-risk patients using prescribing data alone, without spirometry or symptom scores, supporting scalable risk stratification in primary care. This visual framework enables clinicians to stratify patients using only prescribing data, even without algorithmic integration. H: high; L: low; SABA: short-acting β2-agonists; SAMA: short-acting muscarinic antagonists.

Despite its reduced dimensionality, the simplified system maintains robust predictive performance (Figure 2) and offers improved technical and operational feasibility for integration into health information systems and routine care workflows [20-22,43,50,53]. Its lower computational complexity supports real-time patient stratification and personalized COPD management in both primary care and resource-constrained environments (section S6 in Multimedia Appendix 1) [34,38,44].

### Expanded Phenotyping: Comparing Real and Combination Systems

Seleida’s expanded phenotyping system refines the 2025 GOLD ABE classification by incorporating the annual use of SABA or SAMA or both as a stratification axis. Each GOLD category—E (≥2 moderate or ≥1 severe exacerbation), A1/B1 (1 moderate exacerbation), and A0/B0 (no exacerbations)—is subdivided into high SABA users (H; ≥3 canisters per year) and low users (L; <3 canisters per year). This additional layer of segmentation enhances sensitivity for detecting early manifestations of disease instability and symptom burden, yielding a more granular and actionable classification of COPD phenotypes (Table 1).

**Table 1.** Performance metrics of the 126-combination system for each chronic obstructive pulmonary disease phenotype^a^.

	Accuracy, %	Se^b^, %	Sp^c^, %	PPV^d^, %	NPV^e^, %	LR+^f^	LR−	κ value	Significance of κ^g^
A0/B0 H^h^	95.3	100	94.8	66.7	100	19.2	0.00	0.774	<.001^i^
A0/B0 L^j^	96.2	97.7	95.2	93.3	98.4	20.5	0.02	0.922	<.001^i^
A1/B1 H	85.8	50	90.4	40	93.4	5.2	0.55	0.365	.002^k^
A1/B1 L	93.4	81.3	95.6	76.5	96.6	18.3	0.20	0.749	<.001^i^
E H	86.8	38.9	96.6	70	88.5	11.4	0.63	0.431	<.001^i^
E L	97.2	57.1	100	100	97.1	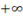	0.43	0.714	<.001^i^

a Sensitivity, specificity, PPV, NPV, and LR+/LR− for each phenotype identified by the 126-combination system. These metrics evaluate the model’s ability to replicate real phenotyping and highlight its strengths in common phenotypes (eg, A0/B0 H and A0/B0 L) as well as areas requiring refinement for less frequent phenotypes (eg, E H and A1/B1 H).

b Se: sensitivity.

c Sp: specificity.

d PPV: positive predictive value.

e NPV: negative predictive value.

f LR: likelihood ratio.

g Significance (*P* value) of Cohen κ coefficient.

h H: high.

i Hypothesis tests applied to assess the statistical significance of the 𝜅 coefficient: Fisher exact test.

j L: low.

k Hypothesis tests applied to assess the statistical significance of the 𝜅 coefficient: χ² test.

Two phenotyping strategies were evaluated: (1) The 126-combination system, designed for high-resolution stratification, is ideally suited to research settings and complex clinical scenarios [27,35]. (2) The simplified 21-combination system, optimized for routine clinical application, offers a favorable balance between predictive performance and computational efficiency [4,39]. Its lower complexity makes it especially appropriate for primary care and resource-limited environments, where operational simplicity and rapid decision-making are essential [43,44].

Both systems exhibited strong concordance with clinician-assigned phenotypes based on real-world data (Cohen κ=0.70, SE=0.050; *P*<.001). Sensitivity and specificity were notably high for the most prevalent phenotypes, with specificity exceeding 90% and sensitivity reaching or surpassing 97% for categories such as A0/B0-H, A0/B0-L, and E-L. In contrast, sensitivity was lower for less frequent phenotypes, underscoring the importance of clinical judgment in borderline cases and reinforcing the complementary role of Seleida alongside expert assessment.

**Figure 3.**
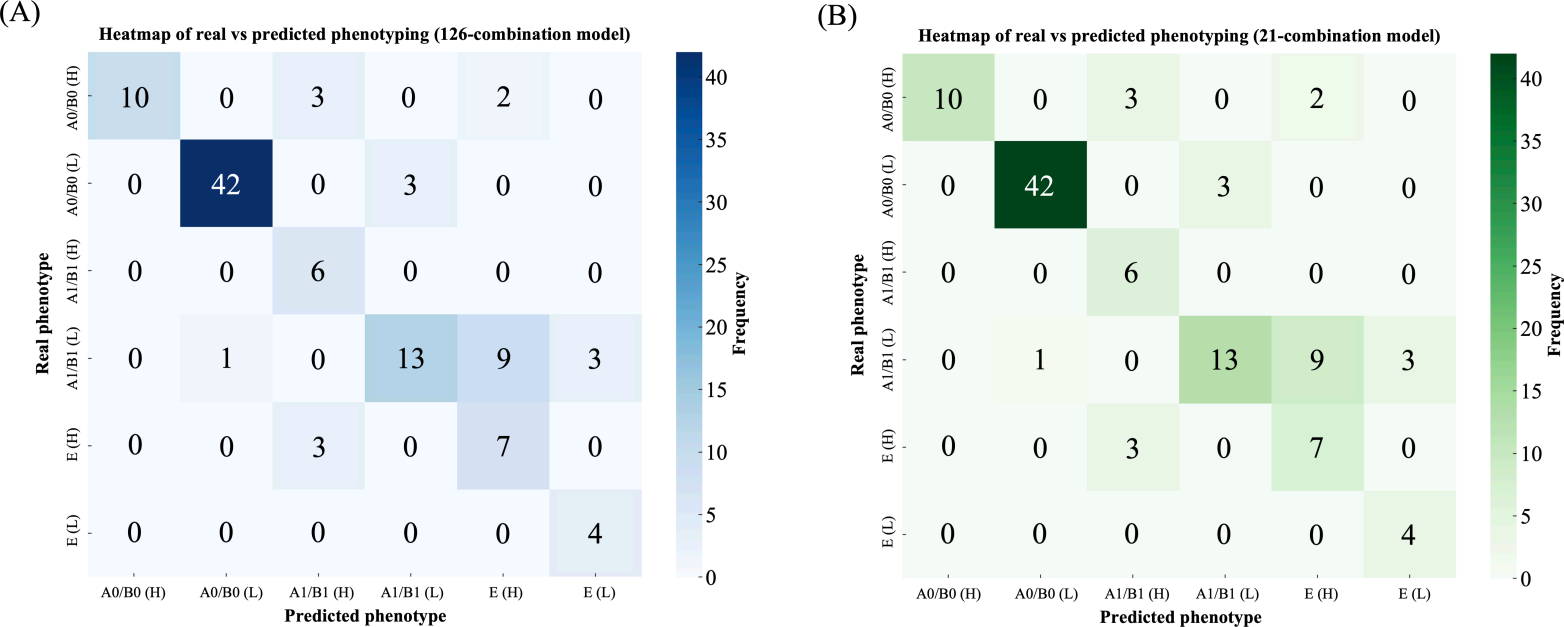
(A) Heatmap of real versus predicted phenotyping by 126-combination system. (B) Heatmap of real versus predicted phenotyping by 21-combination system.

Internal agreement between the 126- and 21-combination models was perfect (Cohen κ=1.00, *P*<.001) (Figure 3A and B). Despite its reduced granularity, the simplified system preserved high specificity, minimizing false positives and confirming its suitability for real-time clinical implementation. These features make it particularly advantageous for integration into EHR systems and point-of-care workflows, especially in settings with constrained health care infrastructure [22,31,43,44,53].

Overall, the expanded phenotyping approach implemented in Seleida offers a scalable, automated solution for enhancing risk stratification and supporting targeted therapeutic interventions in COPD management [20,24,25,38]. By identifying high-risk individuals early, Seleida facilitates more personalized care, prioritizing interventions for those most likely to benefit while reducing overtreatment in stable cases [4,24,25,36]. This paradigm not only improves clinical outcomes but also promotes more efficient use of health care resources—aligning with the growing imperative for data-driven decision-making in contemporary health care systems [21,33,38].

### Basic Phenotyping: Evaluating the 126-Combination System for GOLD 2025 Phenotypes

The performance of Seleida’s 126-combination system was assessed in replicating GOLD 2025 phenotypes [14]—A0/B0 (no exacerbations), A1/B1 (1 moderate exacerbation), and E (≥2 moderate or ≥1 severe exacerbation)—using the annual number of respiratory antibiotic regimens (variable *b*) as the sole predictor (Table 2) [48,49]. The model’s discriminatory capacity across these clinical categories was demonstrated by the following results:

- A0/B0 (no exacerbations): sensitivity=98.1%, specificity=84.9%, and accuracy=97.5%, with near-perfect agreement (κ=0.83; *P*<.001). These findings confirm the model’s high reliability in identifying well-controlled patients.
- A1/B1 (1 moderate exacerbation): sensitivity=67.9%, specificity=83.3%, and accuracy=79.3%, with moderate agreement (κ=0.49; *P*<.001). This level of performance reflects a balanced capacity to detect patients at intermediate risk, despite the inherent clinical heterogeneity of this group.
- E (≥2 moderate or ≥1 severe exacerbation): specificity=96.3%, sensitivity=44%, accuracy=84%, and a high positive LR+=11.9. These metrics indicate robust identification of high-risk patients with minimal false positives, supporting timely and targeted interventions.

**Table 2.** Performance metrics of the 126-combination system for Global Initiative for Chronic Obstructive Lung Disease 2025 chronic obstructive pulmonary disease phenotypes^a^.

	Accuracy, %	Se^b^, %	Sp^c^, %	PPV^d^, %	NPV^e^, %	LR+^f^	LR−	κ value	Significance of κ^g^
A0/B0	97.5	98.1	84.9	86.7	97.8	6.5	0.02	0.830	<.001^h^
A1/B1	79.3	67.9	83.3	59.4	87.8	4.1	0.39	0.489	<.001^i^
E	84	44	96.3	78.6	84.8	11.9	0.58	0.475	<.001^h^

a Sensitivity, specificity, PPV, NPV, LR+ /LR−, accuracy, and к for each phenotype identified by the reverse Seleida model. The metrics reflect the model’s ability to replicate real-world phenotyping and its strengths in classifying Global Initiative for Chronic Obstructive Lung Disease 2025 phenotypes.

b Se: sensitivity.

c Sp: specificity.

d PPV: positive predictive value.

e NPV: negative predictive value.

f LR: likelihood ratios.

g Statistical significance (*P* value) of Cohen к coefficient.

h Hypothesis tests applied to assess the statistical significance of the к coefficient: Fisher exact test.

i Hypothesis tests applied to assess the statistical significance of the к coefficient: χ² test.

Collectively, these results underscore the robustness of Seleida’s basic phenotyping system in stratifying patients based solely on antibiotic prescribing data. However, the model’s ability to discriminate between A and B categories—particularly within the intermediate-risk stratum—may benefit from the incorporation of symptom-based measures, such as the modified Medical Research Council dyspnea scale and the COPD Assessment Test, both of which are widely used in routine practice [16,17,54,55]. Future iterations integrating these variables could enhance phenotypic granularity and improve the model’s adaptability across diverse health care settings.

Despite these acknowledged limitations, Seleida demonstrates strong performance when classifying COPD phenotypes using a single, objective, and consistently recorded variable: annual antibiotic use. The system reliably identifies patients with stable disease (A0/B0), achieves moderate accuracy for those at intermediate risk (A1/B1), and exhibits high specificity for detecting individuals with frequent or severe exacerbations (E). These findings reinforce Seleida’s potential as a pragmatic, evidence-based tool for real-world clinical decision-making, offering scalable, reproducible, and personalized support for COPD management across a wide range of practice environments [29,33,34,38].

### Expanded Phenotyping: Integration of *Pseudomonas* Risk in the 126-Combination System

The 126-combination phenotyping system was further evaluated for its capacity to identify patients with COPD at an increased risk of *Pseudomonas aeruginosa* colonization or infection. Evidence indicates that individuals receiving more than 4 respiratory antibiotic regimens annually exhibit a markedly elevated risk of frequent exacerbations and poor disease control [48,56-58]. By incorporating this threshold into the Seleida model, the system enhances its ability to detect high-risk phenotypic profiles—facilitating targeted interventions such as antimicrobial stewardship and intensified clinical monitoring [48,59].

**Figure 4.**
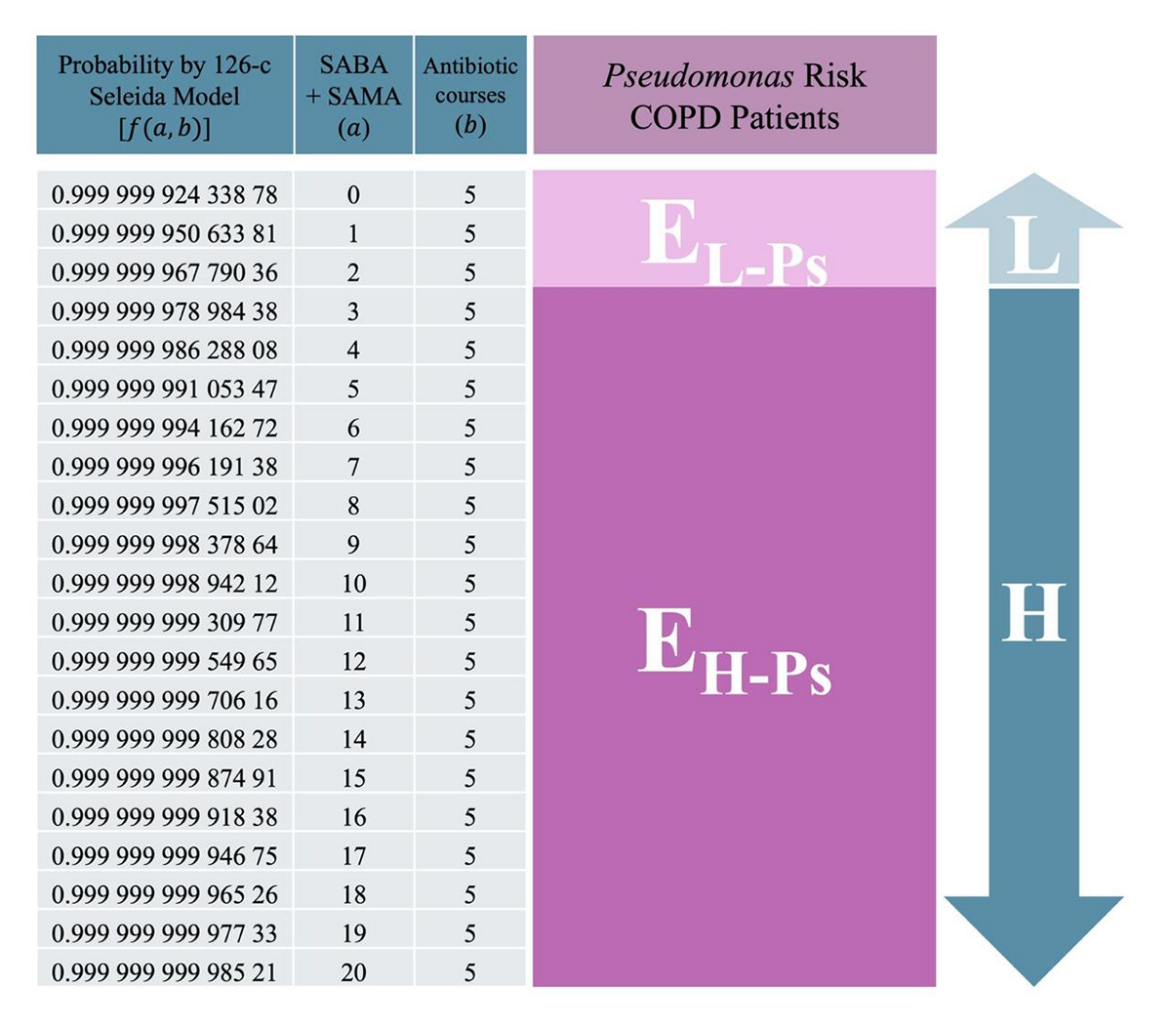
Identification of patients with chronic obstructive pulmonary disease at high risk of *Pseudomonas aeruginosa* colonization using the 126-combination Seleida model. The shaded area highlights phenotypes with high predicted risk (*Pr*≥0.99), ≥5 antibiotics per year (*b*), and high or low rescue inhaler use (H or L). These patients should be prioritized for sputum culture screening and potential referral to pulmonology. This supports targeted infection surveillance based on real-world prescribing data. COPD: chronic obstructive pulmonary disease; H: high; L: low; SABA: short-acting β2-agonists; SAMA: short-acting muscarinic antagonists.

In contrast, the 21-combination system—by design—limits the antibiotic variable (*b*) to a maximum of 2 prescriptions per year, potentially reducing its sensitivity for identifying this severe phenotype. The expanded resolution of the 126-combination system, which accommodates values of *b* up to 5, enables more accurate detection of complex clinical cases. As illustrated in Figure 4, profiles with *b* ≥ 5 are consistently associated with very high predicted risk, capturing severe phenotypes that may otherwise be underrepresented in streamlined models.

Integrating *Pseudomonas*-related risk factors into the high-resolution framework strengthens Seleida’s use for proactive therapeutic planning. This refinement supports the early identification of patients requiring microbiological surveillance, targeted antimicrobial strategies, or referral to hospital-based specialist care—ultimately contributing to the mitigation of antibiotic resistance and the improvement of long-term clinical outcomes (section S6 in Multimedia Appendix 1) [49,60,61].

By capturing a broader spectrum of disease severity, this extension reinforces the adaptability of the Seleida model and underscores its value as a precision tool for guiding personalized interventions in the management of complex COPD phenotypes across a range of clinical environments.

### Impact of Rescue Medication Use on Health Care Utilization in Patients With COPD

High rescue medication use (H) among patients with COPD is a well-established indicator of increased health care resource utilization. In the present cohort (n=106), 71.4% (15/21) of high-use patients required at least 1 respiratory-related clinical consultation annually, compared with 40% (34/85) of low-use patients (*χ*²=6.691; *P*=.010). A moderate yet statistically significant correlation was observed between rescue medication consumption and health care consultations (Pearson *r*=0.251; *P*=.009), as illustrated in Multimedia Appendix 2.

These findings highlight the use of rescue medication as a reliable and actionable marker for patient stratification within the Seleida phenotyping system [25]. Early identification of high-use individuals facilitates the deployment of targeted interventions, including optimization of maintenance therapy, structured education to reduce inhaler overreliance, and scheduling of more frequent clinical follow-ups [15,62,63].

The ≥1 consultation per year threshold serves as a pragmatic and clinically interpretable metric to prioritize high-risk patients in care workflows and inform strategic health care resource allocation. Future analyses incorporating additional parameters—such as disease severity, multimorbidity profiles, and treatment adherence—will be essential to refine predictive accuracy and enhance the model’s capacity to support resource optimization in diverse clinical settings [48,64-66].

### Exacerbations in the Last Year and Rescue Medication Use

In this cohort of 106 patients with COPD, a significant association was observed between exacerbation history and increased use of SABA. Among patients with no exacerbations in the preceding 12 months, 88.7% (47/53) used fewer than 3 rescue canisters annually. In contrast, 28.3% (15/53) of those with ≥1 exacerbation required ≥3 canisters (*χ*²_1_=4.810, *P*=.028; Pearson *r*=0.213, *P=*.028), as depicted in Multimedia Appendix 3.

This correlation underscores the heightened reliance on rescue therapy among patients with recent exacerbations—often reflecting suboptimal maintenance treatment or inadequate symptom control [47,67,68]. These findings support the implementation of targeted clinical strategies, such as optimizing long-term inhaled therapy and reinforcing adherence interventions, to reduce SABA dependence and enhance overall disease management.

Incorporating this association into predictive modeling frameworks can improve risk stratification and enable more personalized treatment planning [69,70]. Future analyses should account for potential confounding factors—such as comorbid conditions, medication adherence, and variability in EHR completeness—to further refine the model’s predictive performance and clinical applicability across heterogeneous patient populations [27,46].

### Treatment Patterns in Patients With Exacerbated COPD

This analysis examined treatment patterns among 53 patients with COPD who experienced at least 1 moderate exacerbation in the past year. After excluding 8 individuals due to missing eosinophil count data, the final sample comprised 45 patients. The Seleida model was applied to assess deviations from current therapeutic recommendations as outlined in established clinical practice guidelines [25].

**Figure 5.**
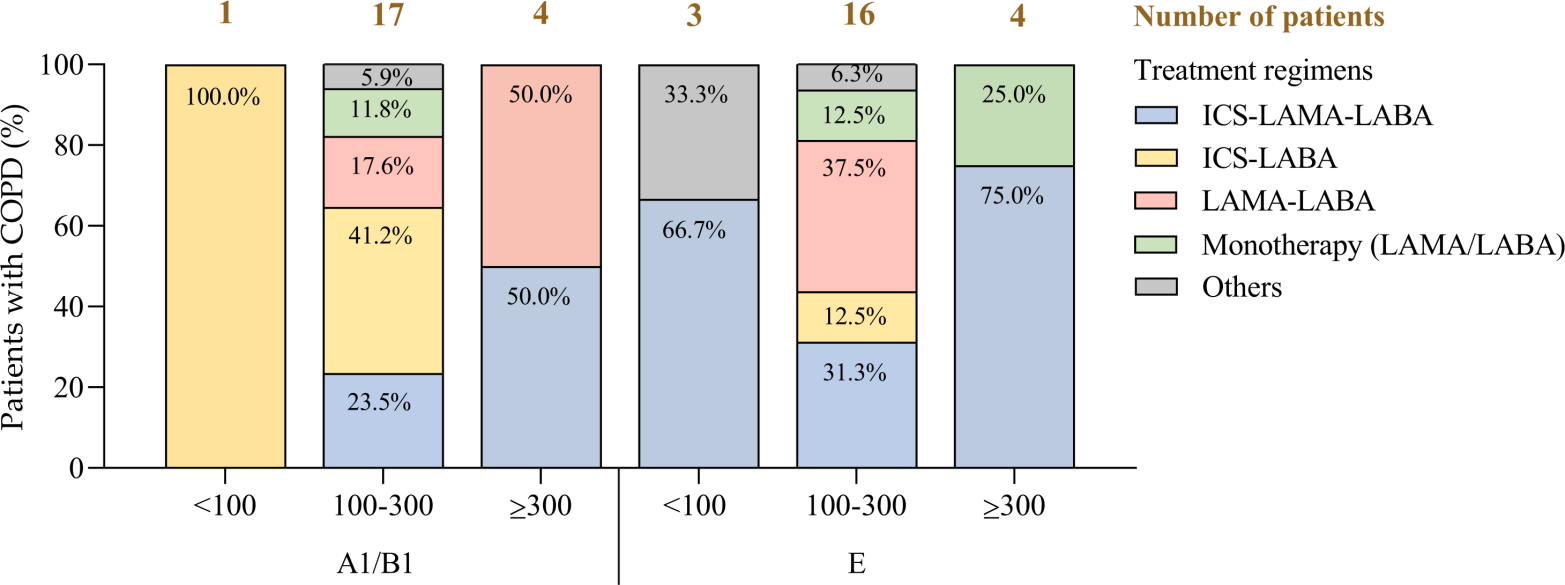
Treatment regimen by annual mean eosinophilia and exacerbation phenotype. COPD: chronic obstructive pulmonary disease; ICS: inhaled corticosteroids; LABA: long-acting β2-agonists; LAMA: long-acting muscarinic antagonists.

Among patients with blood eosinophil counts of <100 cells/µL (4/45, 8.89%)—where the use of inhaled corticosteroids (ICS) is generally not recommended [14]—66.7% (2/3) of those classified as phenotype E, along with 1 patient in the A1/B1 category, were nonetheless prescribed ICS-containing regimens. Exceptions to this pattern were documented in cases where eosinophil counts of ≥100 cells/µL had been recorded previously, justifying continued ICS use (Figure 5). In addition, 11.1% (5/45) of patients were receiving bronchodilator monotherapy, and 22.2% (10/45) were treated with ICS–long-acting β2-agonist combinations, despite GOLD 2025 guidelines recommending dual bronchodilation (long-acting muscarinic antagonist–long-acting β2-agonists) in these clinical scenarios [14].

Conversely, ICS underutilization was observed in patients who met criteria for triple therapy—particularly those with eosinophil counts of ≥100 cells/µL—suggesting that nonclinical factors (eg, logistical or prescriber-related) may contribute to deviations from phenotype-aligned treatment pathways [71].

By automating phenotypic classification based on exacerbation history, the Seleida combination systems—when integrated with eosinophil count and treatment data—allow for the systematic identification of discrepancies between recommended and actual therapy [31,70]. This functionality supports improved alignment with evidence-based guidelines, promoting optimal ICS use, minimizing unnecessary adverse effects, and enhancing overall COPD management outcomes [20,34].

### Real-World COPD Phenotypes Across Daily Inhalation Patterns

**Figure 6.**
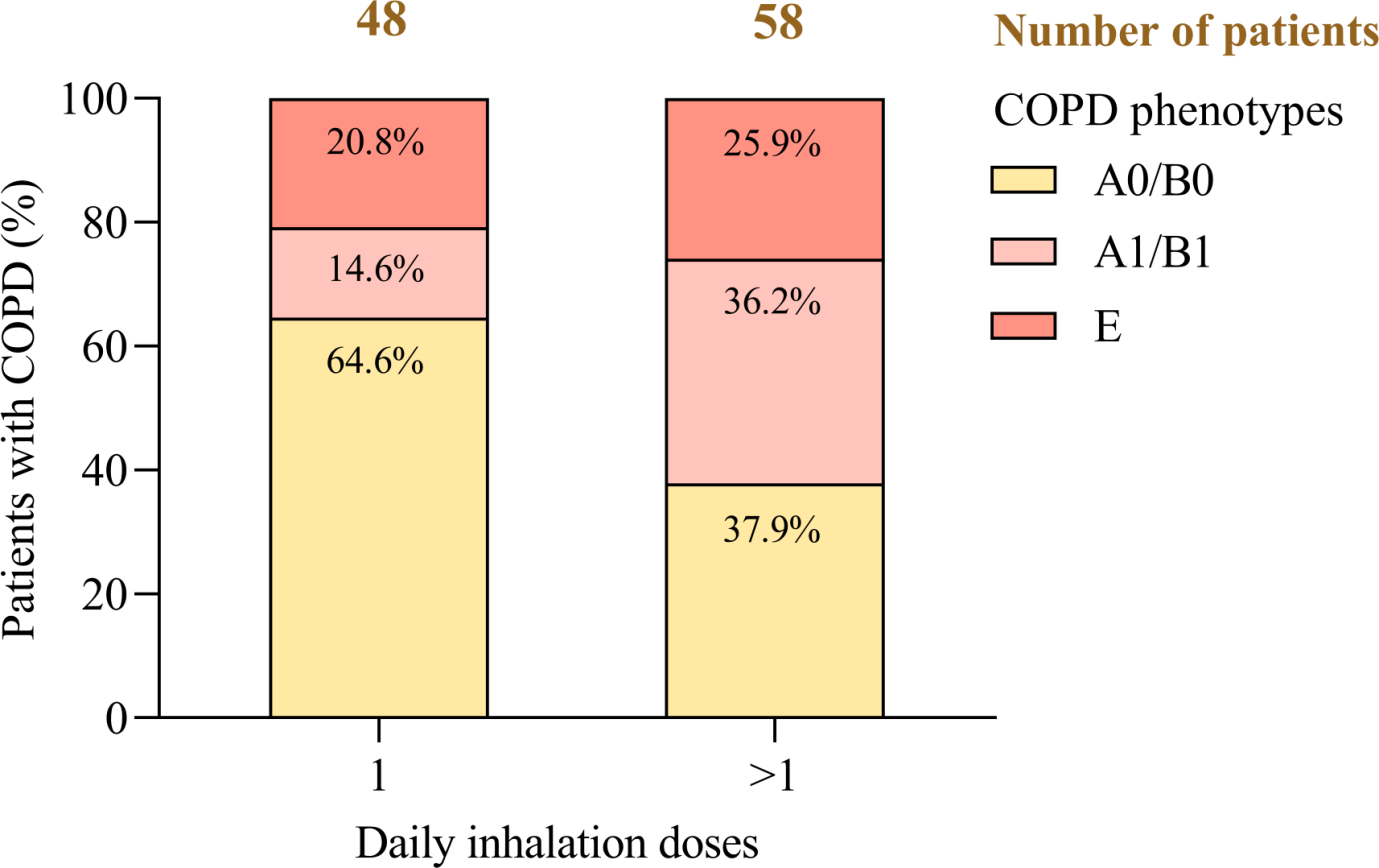
Distribution of chronic obstructive pulmonary disease phenotypes by daily inhalation doses. COPD: chronic obstructive pulmonary disease.

This analysis evaluated the relationship between daily inhalation regimens and COPD phenotypes—A0/B0, A1/B1, and E—in a cohort of 106 patients, examining treatment intensity in relation to clinical severity and exacerbation history (Figure 6).

Among A0/B0 patients (n=53), 58.5% (51/53) required a single inhalation per day, whereas 41.5% (22/53) used 2 or more. In the A1/B1 group (n=28), 75% (21/28) required at least 2 daily inhalations, reflecting higher therapeutic demands. Phenotype E patients (n=25), defined by ≥2 moderate or ≥1 severe exacerbations, exhibited greater variability: 60% (15/25) used 2 or more inhalations daily, while 40% (10/25) managed with a single dose.

Statistical testing revealed a significant association between inhalation frequency and clinical phenotype (*χ*²_²_=8.662; *P*=.013). A linear trend (*P*=.048), alongside weak but statistically significant correlations (Pearson *r*=0.193, *P*=.047; Spearman *r*=0.215, *P*=.027), suggests that patients with higher-risk phenotypes tend to receive more intensive inhalation regimens.

These findings underscore the relevance of phenotype-guided treatment strategies: while low-intensity regimens may suffice for A0/B0 patients, individuals classified as A1/B1 or E typically require escalation of therapy. Incorporating daily inhalation patterns into predictive models enhances clinical personalization, supports adherence to guideline-based care, and contributes to improved patient outcomes—advancing the principles of precision medicine in COPD management [70].

### Real-World COPD Phenotypes and Health Care Utilization

**Figure 7.**
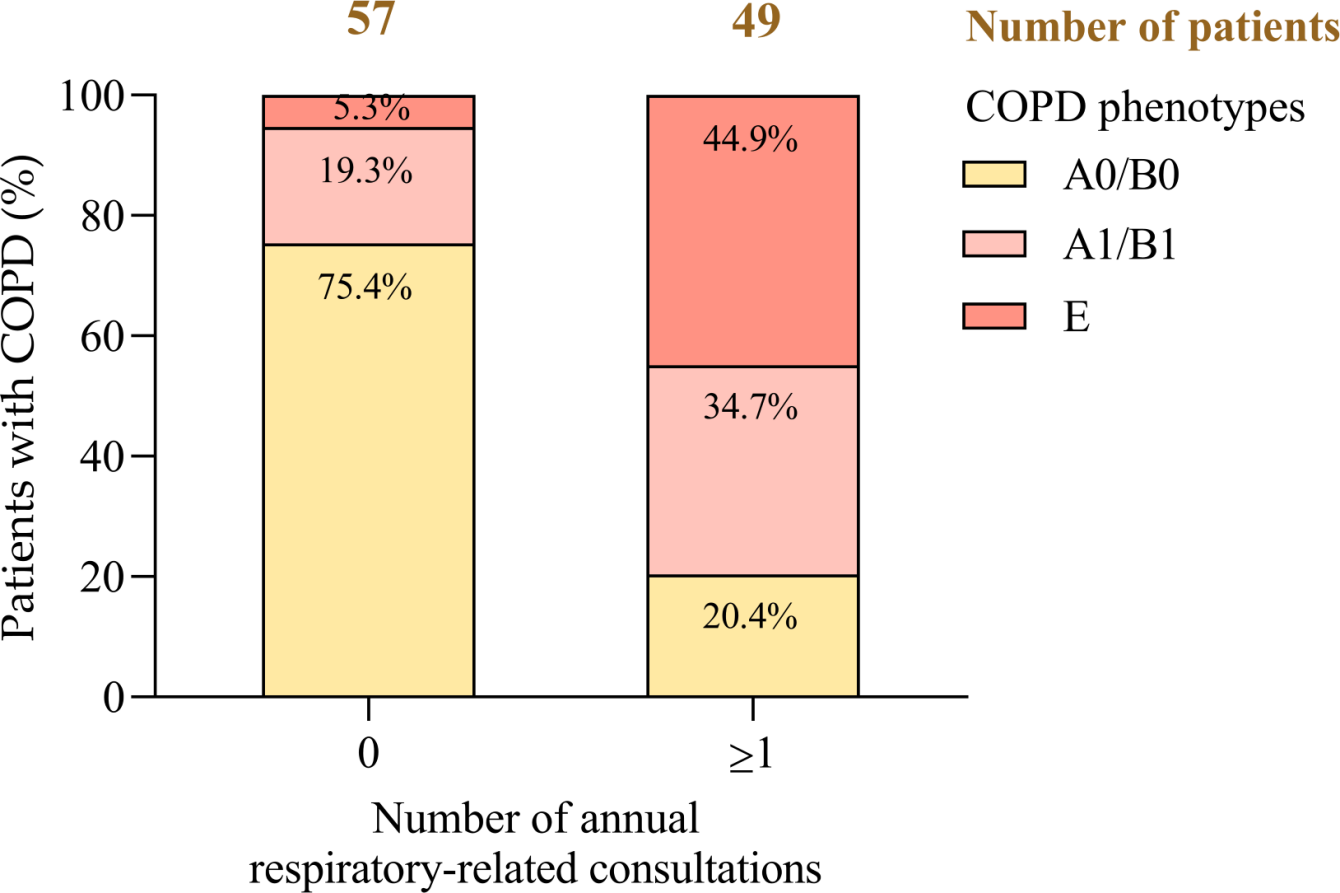
Proportion of chronic obstructive pulmonary disease phenotypes by number of annual health care consultations. COPD: chronic obstructive pulmonary disease.

This analysis examined the association between COPD phenotypes—A0/B0, A1/B1, and E—and respiratory-related health care utilization in a cohort of 106 patients, with a focus on the need for at least 1 annual clinical consultation (Figure 7).

- A0/B0 phenotype (n=53): 81.1% (43/53) of patients required no respiratory-related consultations, while 18.9% (10/53) needed at least 1, reflecting stable disease and low health care demand.
- A1/B1 phenotype (n=28): 60.7% (17/28) required at least 1 consultation, indicating increased follow-up needs linked to a history of moderate exacerbations.
- E phenotype (n=25): 88% (22/25) required at least 1 consultation, representing the highest level of health care utilization, consistent with more severe disease and frequent exacerbations.

Statistical analysis confirmed a robust association between phenotype and health care utilization (*χ*²_²_=35.873; *P<*.001), with strong, statistically significant correlations observed (Pearson *r*=0.578; Spearman *r*=0.582; both *P<*.001).

These findings demonstrate that health care resource demand increases in parallel with disease severity—ranging from minimal utilization in patients with stable phenotypes (A0/B0) to substantially elevated needs in those classified as phenotype E. Integrating phenotype-based stratification into clinical workflows can support more efficient resource allocation, ensuring timely intervention for high-risk patients while minimizing unnecessary consultations among those with controlled disease.

## Discussion

### Principal Findings

This study presents and internally validates Seleida—a fully automated, deterministic, and bijective model designed to assess COPD control and generate clinically interpretable phenotypes using structured EHR data. By relying exclusively on routinely recorded prescribing information—specifically, the annual number of dispensed SABA or SAMA canisters or both and respiratory antibiotics—Seleida offers a scalable and transparent alternative to conventional classification frameworks such as GOLD 2025 and GesEPOC 2021. These frameworks often depend on intermittently documented spirometry results and symptom scores, limiting their feasibility and consistency in real-world, especially primary care, settings [14,17,18,47,52,72].

The decision to implement LASSO over Ridge or Elastic Net in the Seleida model was based on a comparative analysis of regularization techniques. While all 3 approaches demonstrated acceptable discrimination, LASSO provided the best balance between predictive accuracy, parsimony, and clinical interpretability. Ridge regression tended to overshrink coefficients, whereas Elastic Net offered no additional benefit in a low-dimensional, low-collinearity setting (section S4 in Multimedia Appendix 1). This methodological choice reinforces Seleida’s role as a computationally efficient and clinically actionable tool for phenotype-guided COPD management.

The model demonstrated perfect agreement between its 126- and 21-combination phenotyping schemas (Cohen κ=1.00; *P<*.001) and substantial concordance with clinician-assigned phenotypes based on GOLD 2025 criteria (κ=0.70; *P<*.001), confirming its internal robustness and interpretive reliability [42,73]. By leveraging high-frequency, objective EHR variables, Seleida enables dynamic risk stratification even in the absence of structured clinical assessments—particularly relevant in settings where such data may be unavailable or inconsistently applied.

Given that nearly half of individuals with COPD experience at least 1 moderate to severe exacerbation annually—often without being flagged as uncontrolled by current tools—Seleida addresses a critical gap by providing continuous, data-driven risk stratification embedded within routine care pathways [4,5].

To date, no automated or EHR-integrated phenotyping model has been formally established for COPD in real-world clinical settings. Unlike existing unidirectional risk scores or machine learning classifiers focused on exacerbation or mortality risk, Seleida was explicitly designed as a bidirectional and bijective system tailored to primary care populations. This architecture enables both forward prediction from observed data and reverse inference of phenotypic profiles from computed probabilities—features essential for transparency, auditability, and integration into decision support environments.

Crucially, the model relies solely on structured variables that are universally available across health systems, ensuring interoperability and scalability. As such, benchmark comparisons against nonbijective models were deemed conceptually misaligned and methodologically uninformative. Instead, internal comparisons were performed (section S4 in Multimedia Appendix 1), and the final implementation was selected based on predictive accuracy, sparsity, and operational interpretability.

To the best of the authors’ knowledge—based on an exhaustive PubMed search using combinations of terms such as COPD, automatic phenotyping, clinical decision support systems, electronic health records, predictive modeling, algorithmic classification, bijective model, and rule-based phenotyping—no published model currently exists that is specifically designed for the automatic phenotyping of patients with COPD and suitable for direct integration into health care information systems. Future studies may explore head-to-head evaluations once externally validated phenotyping frameworks become available.

### Implications for Digital Health Integration

Seleida was designed to overcome long-standing barriers to the adoption of clinical decision support tools, including limited data availability, platform heterogeneity, and clinician disengagement driven by system opacity or poor integration. Its deterministic and parsimonious architecture ensures full traceability and facilitates seamless integration across heterogeneous digital infrastructures. The model’s implementation potential can be articulated across 3 core domains:

- EHR integration and technical feasibility: Seleida requires only 2 structured input variables—annual dispensations of SABA or SAMA canisters or both and respiratory antibiotics—which are consistently recorded in most EHRs. Its architecture adheres to HL7 FHIR standards, as detailed in section S7 in Multimedia Appendix 1, where all model outputs were successfully validated using the official HL7 FHIR validator (v6.5.28) without structural or semantic errors across 440 resources. These outputs were generated through an automated simulation of valid (*a, b*) input combinations—not from real patient data—and were used to demonstrate the model’s technical interoperability under HL7 FHIR specifications. The structured Fast Healthcare Interoperability Resources (FHIR) bundles include *RiskAssessment* (risk and rationale), *DetectedIssue* (clinical recommendations), *Condition* (disease state), and *Provenance* (model metadata), ensuring syntactic integrity, semantic traceability, and compatibility with clinical decision support platforms.
- Clinical decision support integration: The generated FHIR bundles enable real-time deployment within EHR-based dashboards and alert systems via standard Application Programming Interfaces. For instance, exceeding predefined thresholds (eg, >3 inhalers per year or ≥2 antibiotic courses) can trigger automated clinical recommendations through the *DetectedIssue* resource, which remains fully auditable via associated *Provenance* metadata. This architecture supports reproducible and scalable implementation in both open-source platforms (eg, OpenMRS) and commercial systems compliant with HL7 R5.
- Scalability and global interoperability: The model’s minimal data requirements and exclusive reliance on universally structured prescribing variables make it well suited for adoption in low- and middle-income countries. Its lightweight, FHIR-compliant design allows integration into national health information infrastructures aligned with the World Health Organization’s Digital Health Strategy. The use of international terminologies (SNOMED CT and LOINC) and standardized value sets further enables longitudinal data integration, federated learning, and interoperability across certified clinical environments worldwide.

### Advancing Precision COPD Management

Seleida contributes to the advancement of precision medicine in COPD by enabling automated, phenotype-driven decision pathways tailored to real-world primary care data. Its deterministic architecture supports individualized care planning through multiple mechanisms. First, the model’s integration of 2025 GOLD ABE classification with SABA dependency stratification facilitates personalized pharmacologic strategies—informing step-up or step-down adjustments based on quantified risk levels. Beyond pharmacologic guidance, Seleida helps identify patients who may benefit from nonpharmacologic interventions such as structured pulmonary rehabilitation or smoking cessation support, reinforcing a holistic, patient-centered approach to disease control [74,75]. Moreover, the ability to isolate phenotypes associated with frequent exacerbations or suggestive of *P aeruginosa* colonization offers additional value for microbiological surveillance and early antimicrobial stewardship [76].

Seleida’s modular structure permits future expansion. Variables such as comorbidity profiles, vaccination status, or inflammatory biomarkers (eg, eosinophil count and C-reactive protein) can be incorporated without compromising interpretability—enhancing predictive precision and adaptability to diverse clinical settings.

### Health System Optimization and Economic Potential

Beyond individual risk estimation, Seleida generates population-level insights that can inform health system optimization and performance monitoring. Its probabilistic outputs enable benchmarking of COPD control across institutions and regions, helping identify care gaps and guiding policy interventions to reduce diagnostic inertia, undertreatment, and geographic disparities in disease management.

At the public health level, the model supports strategic planning by quantifying the projected impact of interventions—such as seasonal vaccination campaigns, air quality alerts, or therapeutic adjustments—on exacerbation rates and control metrics at scale. Its forecasting capacity also informs resource allocation, enabling estimates of emergency visits, hospitalization demands, and inhaler supply needs—particularly critical in overstretched systems or during seasonal peak periods.

From an economic perspective, COPD exacerbations—especially those requiring hospitalization—account for more than 70% of direct disease-related health care expenditures [77]. By identifying high-risk patients early, Seleida may help reduce exacerbation incidence by approximately 15%‐25%, consistent with reductions observed in phenotype-guided management programs [74,75]. Preventing a single severe exacerbation may yield direct savings of $1700 to $4720 (the cost that was originally reported in euros [€] was converted to US dollars using the exchange rate of €1=$1.18 as of September 15, 2025, based on the European Central Bank reference rates per patient annually) [21]. While these projections remain hypothetical within Seleida’s current context, they underscore its potential contribution to value-based care. Confirmatory evidence from prospective cost-effectiveness evaluations in multicenter implementation studies will be essential to fully quantify this impact.

As health care systems face mounting pressure to improve outcomes while managing resource constraints, tools such as Seleida—anchored in objective, high-frequency data and deployable across diverse infrastructures—offer a pragmatic path toward more efficient, equitable, and data-driven respiratory care.

### Interpretability and Clinical Use in Real-World Practice

Seleida was designed for seamless integration into EHR systems, where structured clinical inputs—specifically rescue inhaler use and antibiotic prescriptions—can be continuously analyzed to generate real-time risk estimates and automated alerts. The model can also be applied manually in settings without digital integration, allowing clinicians to interpret the probability of poor control as a continuous indicator of exacerbation risk, with higher values reflecting proportionally greater clinical vulnerability. This dual deployment capacity is particularly valuable in resource-constrained environments where decision support infrastructures are limited.

For exploratory implementation, a provisional risk threshold was established. Because the sample size of this pilot study precluded formal statistical derivation, a reference point of *Pr*>.50 was selected based on the internal distribution of predicted probabilities. This should be interpreted not as a strict clinical cutoff but as an operational inflection point to guide prioritization. The model supports graded stratification across the risk spectrum, enabling providers to flag higher-risk profiles (eg, *Pr*>.70) for proactive evaluation, treatment adjustment, or closer follow-up.

Beyond individual patient care, Seleida enables population-level case finding using prescribing data alone, without requiring recent consultations or the patient’s physical presence. This capability allows the generation of risk-stratified reports for entire patient panels, facilitating earlier identification of those likely to be poorly controlled. The transition from reactive recognition during encounters to anticipatory detection at the database level supports a scalable, equitable, and resource-efficient approach to COPD management. By combining point-of-care guidance with population surveillance, Seleida offers a flexible solution adaptable to diverse health care settings.

From a clinical perspective, fully automated and bijective phenotyping enables early detection of unstable COPD without the need for spirometry or symptom scores. By generating actionable, guideline-aligned classifications from routine EHR data, Seleida supports timely treatment selection, targeted microbiological assessment, and precise referral. Prioritizing follow-up for poorly controlled patients has the potential to reduce primary care and emergency visits, exacerbations, hospitalizations, and even mortality, while improving disease control and optimizing health care resource use.

Seleida’s deterministic and bijective framework delivers precise, phenotype-driven COPD management using only routinely collected prescribing data. By translating high-frequency medication patterns into actionable risk profiles, it facilitates earlier therapeutic escalation, targeted treatment stratification, and proactive follow-up scheduling—particularly in patients who might otherwise remain undetected by conventional spirometry-based or symptom-based approaches.

The model’s simplicity, transparency, and full EHR interoperability enable smooth integration into clinical workflows, supporting earlier detection of instability, automated triage, and efficient resource allocation. At the system level, Seleida empowers population-wide surveillance, equitable access to timely interventions, and optimization of health care resources—priorities central to contemporary primary care and health system planning.

At the health system level, proactive, database-driven detection enables earlier triage and strategic resource allocation across the care continuum. Identifying high-risk patients before clinical deterioration allows services to intensify follow-up, focus preventive interventions, and streamline emergency response. This anticipatory approach promotes equitable access to timely care, reduces unnecessary utilization, and decreases the likelihood of preventable hospitalizations—enhancing the efficiency, scalability, and sustainability of COPD management while maximizing the effectiveness of interventions in primary care.

### Limitations and Validation Needs

Despite its strengths, several limitations warrant consideration:

- Sample size and scope: The model was validated retrospectively in a single-country cohort of 106 patients from 2 Spanish primary care centers. While the results support internal consistency, the generalizability to other health systems, demographic groups, and care models remains to be established through multicenter external validation [45]. To address this, future validation studies are planned with larger and more heterogeneous cohorts drawn from multiple Spanish regions, aiming to evaluate the model’s performance across broader demographic, geographic, and clinical contexts. Given its reliance on universally recorded prescribing data, Seleida also has strong potential for applicability in diverse international settings, and validation efforts will extend to health care systems in both European and non-European countries.
- Variable parsimony: Seleida was intentionally designed for simplicity and scalability, relying on 2 objective, high-frequency prescribing variables. However, this design may limit sensitivity in clinically complex or multimorbid phenotypes. In particular, the model may underperform in identifying atypical exacerbation profiles, discordant symptom patterns, or overlapping syndromes such as asthma-COPD overlap syndrome, eosinophilic COPD, or symptom-dominant cases with low exacerbation burden [78].
- Fixed thresholds and model rigidity: The deterministic and bijective architecture of Seleida requires predefined integer inputs (*a, b*), which enhances reproducibility and transparency but may reduce flexibility. This could limit performance in borderline or transitional cases, especially in patients with fluctuating clinical trajectories near decision thresholds. Future iterations may incorporate hybrid models with continuous or adaptive inputs, leveraging machine learning–based adjustments to enhance model responsiveness and flexibility [79].
- Omission of conventional clinical metrics: The exclusion of spirometry (eg, forced expiratory volume in the first second of expiration), symptom scores (eg, COPD Assessment Test, modified medical research council, and patient-reported outcomes enhances operational feasibility but may reduce clinical acceptability in settings where such metrics are routinely used for monitoring, reimbursement, or treatment authorization. Furthermore, Seleida may not fully capture disease burden in patients whose impact is driven more by symptoms than by exacerbation frequency—highlighting the need for future multimodal extensions.

### Future Directions

To support broader adoption, equity, and scientific transparency, several development paths are under consideration:

1. Multinational validation: Future collaborations with international partners—such as the WHO Digital Health Programme—may enable cross-context evaluation of Seleida using open-source EHR infrastructures such as OpenMRS. This would facilitate assessment in diverse health care systems, including low- and middle-income settings.
2. Artificial intelligence–enhanced predictive modeling: Planned extensions include the incorporation of machine learning components to enable dynamic thresholds, time series forecasting, and the integration of social and environmental determinants of health. These enhancements aim to improve contextual relevance, longitudinal accuracy, and model adaptability in complex clinical scenarios.
3. Open-science reproducibility: A complete technical framework—including public, detailed model documentation, and executable pseudocode—could be released to support independent replication, collaborative development, and global dissemination. This approach would follow successful precedents in predictive modeling platforms, such as QRISK [80].

### Ethical Considerations and Equity

Future iterations will incorporate algorithmic fairness audits to detect and mitigate potential biases in risk estimation—particularly among historically underrepresented or underserved populations. Equity by design is a foundational principle of the Seleida framework. By relying exclusively on structured prescribing data—universally recorded across health systems—and remaining independent of costly diagnostic tools or patient-reported inputs, Seleida is inherently scalable and adaptable to low-resource settings. Its compatibility with open-source EHR infrastructures, such as OpenMRS, facilitates national-scale deployment in both high- and middle-income countries. Partnerships with public health authorities and patient advocacy groups are envisioned to ensure that Seleida promotes access and actively reduces—rather than reinforces—structural disparities in COPD management [81-84].

**Figure 8.**
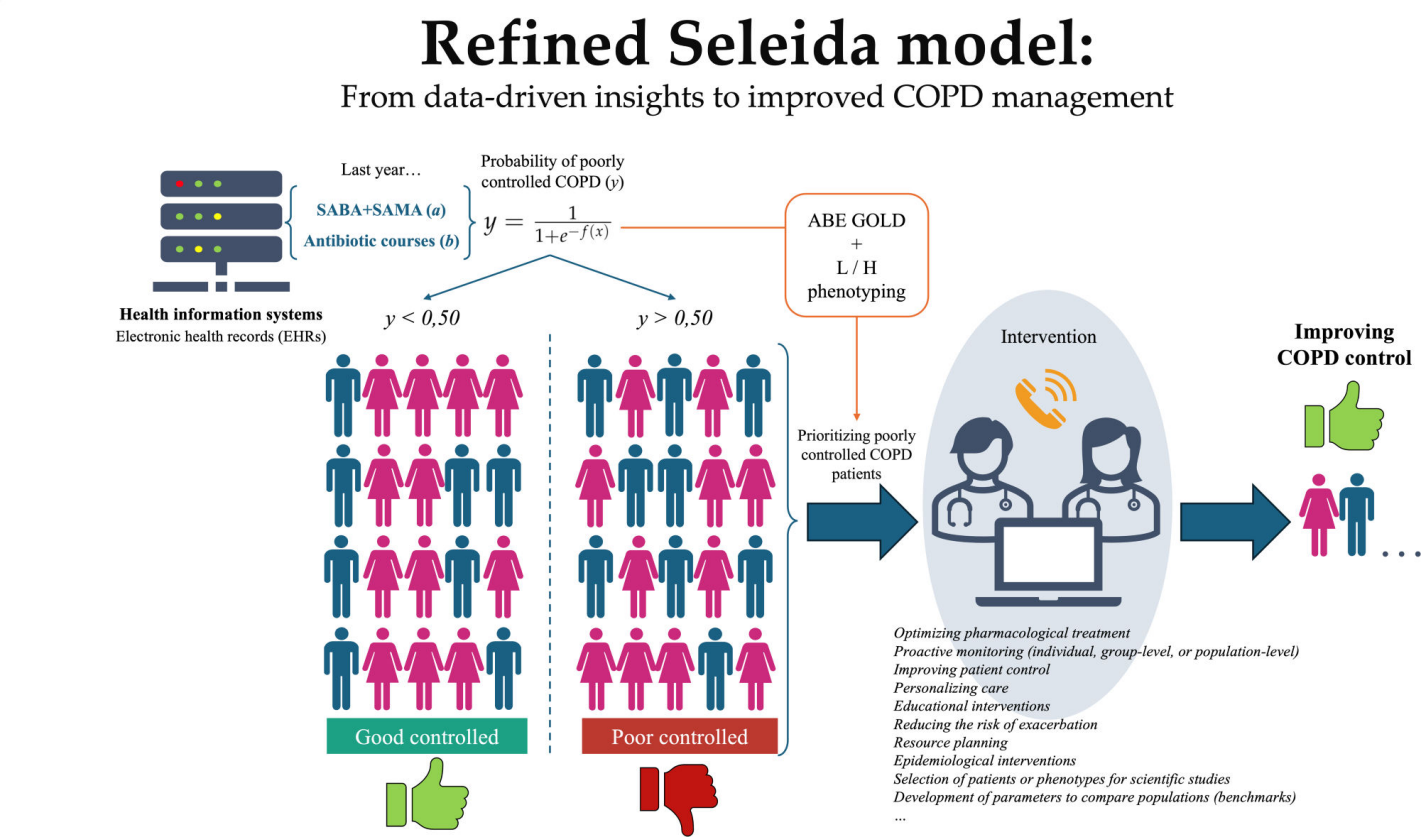
Applications of the refined Seleida model. The model uses EHR data to identify patients with poorly controlled chronic obstructive pulmonary disease, enabling phenotyping and targeted interventions to improve disease management. COPD: chronic obstructive pulmonary disease; EHR: electronic health care record; GOLD: Global Initiative for Chronic Obstructive Lung Disease; H: high; L: low; SABA: short-acting β2-agonists; SAMA: combined with short-acting muscarinic antagonists.

By bridging predictive analytics with frontline clinical decision-making, Seleida supports equitable, individualized care (Figure 8). Its automated, interoperable, and fully transparent architecture enables the early identification of poorly controlled patients and the delivery of actionable, risk-aligned interventions. Through the transformation of routine prescribing data into clinically interpretable phenotypes, Seleida contributes not only to personalized medicine but also to system-level optimization and population risk mitigation.

Planned multicenter validation and open-science dissemination are expected to further reinforce Seleida’s role as a cornerstone in the emerging field of data-driven respiratory precision medicine—advancing a vision grounded in accessibility, transparency, and global equity.

### Conclusions

This study presents Seleida as the first model for assessing COPD control that is fully automated, mathematically bijective, and clinically validated using structured data routinely captured in primary care. By leveraging 2 objective, high-frequency prescribing variables—namely, the number of dispensed rescue inhaler canisters and respiratory antibiotic courses—Seleida enables scalable, reproducible, and clinically interpretable phenotyping aligned with real-world care workflows.

The model demonstrated strong internal validity, with perfect concordance between its high-resolution and streamlined phenotyping systems (κ=1.00; *P<*.001), and substantial agreement with clinician-assigned phenotypes based on GOLD 2025 criteria (κ=0.70; *P<*.001), supporting both its discriminative capacity and interpretive reliability. Its deterministic structure supports real-time risk estimation and reverse phenotype mapping, enabling individualized treatment planning within EHR-integrated environments.

Designed for seamless digital integration, Seleida’s low data burden and full compatibility with HL7 FHIR standards facilitate implementation across a broad range of informatics platforms—including dashboards, telehealth systems, and registry-based surveillance tools. These features, along with its transparency and interoperability, make the model particularly applicable to resource-limited settings.

While the present validation was limited to a retrospective cohort from 2 Spanish primary care centers, prospective multicenter studies are underway to evaluate Seleida’s generalizability, economic impact, and performance across diverse health systems. Future model iterations aim to enhance flexibility and contextual precision through adaptive thresholds, multimodal inputs, and algorithmic fairness auditing.

By transforming routinely collected prescribing data into actionable, risk-aligned insights, Seleida offers a reproducible and ethically grounded approach to phenotype-guided COPD care. It provides a concrete, scalable contribution toward operationalizing precision medicine principles in everyday respiratory practice.

## Supplementary material

10.2196/74932 Multimedia Appendix 1 Supplementary methods and results (sections S1-S7).

10.2196/74932 Multimedia Appendix 2 Health care consultations by rescue medication use (L/H). COPD: chronic obstructive pulmonary disease; H: high; L: low.

10.2196/74932 Multimedia Appendix 3 Exacerbations in the last year by rescue medication use (L/H). COPD: chronic obstructive pulmonary disease; H: high; L: low.

## Abbreviations

COPD: chronic obstructive pulmonary disease
EHR: electronic health record
FHIR: Fast Healthcare Interoperability Resources
GOLD: Global Initiative for Chronic Obstructive Lung Disease
H: high
HL7 FHIR: Health Level Seven Fast Healthcare Interoperability Resources
ICD-10: International Classification of Diseases, Tenth Revision
ICS: inhaled corticosteroid
L: low
LABA: Long-acting β2-agonists
LAMA: Long-acting muscarinic antagonists
LASSO: Least Absolute Shrinkage and Selection Operator
LR: likelihood ratio
ML: machine learning
NPV: Negative predictive value
PPV: Positive predictive value
Pr: Probability of poor control
SABA: short-acting β2-agonists
SAMA: short-acting muscarinic antagonists
Se: Sensitivity
Sp: Specificity

## References

[1] Boers E, Barrett M, Su JG (2023). Global burden of chronic obstructive pulmonary disease through 2050. JAMA Netw Open.

[2] GBD 2019 Chronic Respiratory Diseases Collaborators (2023). Global burden of chronic respiratory diseases and risk factors, 1990-2019: an update from the Global Burden of Disease Study 2019. EClinicalMedicine.

[3] Harries TH, White P (2021). Spotlight on primary care management of COPD: electronic health records. Chron Respir Dis.

[4] Sandelowsky H, Weinreich UM, Aarli BB (2021). COPD—do the right thing. BMC Fam Pract.

[5] Jones RCM, Price D, Ryan D (2014). Opportunities to diagnose chronic obstructive pulmonary disease in routine care in the UK: a retrospective study of a clinical cohort. Lancet Respir Med.

[6] Joo MJ, Au DH, Fitzgibbon ML, McKell J, Lee TA (2011). Determinants of spirometry use and accuracy of COPD diagnosis in primary care. J Gen Intern Med.

[7] Joo MJ, Sharp LK, Au DH, Lee TA, Fitzgibbon ML (2013). Use of spirometry in the diagnosis of COPD: a qualitative study in primary care. COPD.

[8] Price DB, Yawn BP, Jones RCM (2010). Improving the differential diagnosis of chronic obstructive pulmonary disease in primary care. Mayo Clin Proc.

[9] Daniels K, Lanes S, Tave A (2024). Risk of death and cardiovascular events following an exacerbation of COPD: the EXACOS-CV US Study. Int J Chron Obstruct Pulmon Dis.

[10] Hawkins NM, Nordon C, Rhodes K (2024). Heightened long-term cardiovascular risks after exacerbation of chronic obstructive pulmonary disease. Heart.

[11] Zysman M, Nordon C, Fabry-Vendrand C (2025). Risk of cardiovascular events according to the severity of an exacerbation of chronic obstructive pulmonary disease. Eur J Prev Cardiol.

[12] Nordon C, Simons SO, Marshall J (2025). The sustained increase of cardiovascular risk following COPD exacerbations: meta-analyses of the EXACOS-CV studies. ERJ Open Res.

[13] Singh H, Schiff GD, Graber ML, Onakpoya I, Thompson MJ (2017). The global burden of diagnostic errors in primary care. BMJ Qual Saf.

[14] (2025). Global strategy for the diagnosis, management, and prevention of chronic obstructive pulmonary disease. https://goldcopd.org/wp-content/uploads/2024/11/GOLD-2025-Report-v1.0-15Nov2024_WMV.pdf.

[15] Miravitlles M, Calle M, Molina J (2022). Spanish COPD Guidelines (GesEPOC) 2021: updated pharmacological treatment of stable COPD. Arch Bronconeumol.

[16] Miravitlles M, Soler-Cataluña JJ, Calle M (2017). Spanish COPD Guidelines (GesEPOC) 2017. Pharmacological treatment of stable chronic obstructive pulmonary disease. Arch Bronconeumol.

[17] Cosío BG, Hernández C, Chiner E (2022). [Translated article] Spanish COPD Guidelines (GesEPOC 2021): non-pharmacological treatment update. Arch Bronconeumol.

[18] Gondalia R, Bender BG, Theye B, Stempel DA (2019). Higher short-acting beta-agonist use is associated with greater COPD burden. Respir Med.

[19] Rockenschaub P, Jhass A, Freemantle N (2020). Opportunities to reduce antibiotic prescribing for patients with COPD in primary care: a cohort study using electronic health records from the Clinical Practice Research Datalink (CPRD). J Antimicrob Chemother.

[20] Zeng S, Arjomandi M, Tong Y, Liao ZC, Luo G (2022). Developing a machine learning model to predict severe chronic obstructive pulmonary disease exacerbations: retrospective cohort study. J Med Internet Res.

[21] Braa J, Hanseth O, Heywood A, Mohammed W, Shaw V (2007). Developing health information systems in developing countries: the flexible standards strategy. MIS Q.

[22] Shull JG (2019). Digital health and the state of interoperable electronic health records. JMIR Med Inform.

[23] Booth HP, Gallagher AM, Mullett D (2019). Quality improvement of prescribing safety: a pilot study in primary care using UK electronic health records. Br J Gen Pract.

[24] Navarro Ros FM, Maya Viejo JD (2024). Preclinical evaluation of electronic health records (EHRs) to predict poor control of chronic respiratory diseases in primary care: a novel approach to focus our efforts. J Clin Med.

[25] Maya Viejo JD, Navarro Ros FM (2025). Preclinical identification of poorly controlled COPD: patients with a single moderate exacerbation matter too. J Clin Med.

[26] Marovic B, Curcin V (2020). Impact of the European General Data Protection Regulation (GDPR) on health data management in a European Union candidate country: a case study of Serbia. JMIR Med Inform.

[27] Weiskopf NG, Weng C (2013). Methods and dimensions of electronic health record data quality assessment: enabling reuse for clinical research. J Am Med Inform Assoc.

[28] Sterne JAC, White IR, Carlin JB (2009). Multiple imputation for missing data in epidemiological and clinical research: potential and pitfalls. BMJ.

[29] Peduzzi P, Concato J, Kemper E, Holford TR, Feinstein AR (1996). A simulation study of the number of events per variable in logistic regression analysis. J Clin Epidemiol.

[30] Palojoki S, Saranto K, Reponen E, Skants N, Vakkuri A, Vuokko R (2021). Classification of electronic health record-related patient safety incidents: development and validation study. JMIR Med Inform.

[31] Mandel JC, Kreda DA, Mandl KD, Kohane IS, Ramoni RB (2016). SMART on FHIR: a standards-based, interoperable apps platform for electronic health records. J Am Med Inform Assoc.

[32] FHIR overview. HL7 FHIR Release 5.

[33] Canova-Barrios C, Machuca-Contreras F (2022). Interoperability standards in Health Information Systems. Semin Med Writ Educ.

[34] Trinkley KE, Kroehl ME, Kahn MG (2021). Applying clinical decision support design best practices with the practical robust implementation and sustainability model versus reliance on commercially available clinical decision support tools: randomized controlled trial. JMIR Med Inform.

[35] Pikoula M, Quint JK, Nissen F, Hemingway H, Smeeth L, Denaxas S (2019). Identifying clinically important COPD sub-types using data-driven approaches in primary care population based electronic health records. BMC Med Inform Decis Mak.

[36] Halpin DMG, Miravitlles M, Metzdorf N, Celli B (2017). Impact and prevention of severe exacerbations of COPD: a review of the evidence. Int J Chron Obstruct Pulmon Dis.

[37] Saini V, Technical S, Manager P (2022). Data quality assurance strategies in interoperable health systems. J Artif Intell Res.

[38] Sutton RT, Pincock D, Baumgart DC, Sadowski DC, Fedorak RN, Kroeker KI (2020). An overview of clinical decision support systems: benefits, risks, and strategies for success. NPJ Digit Med.

[39] Collins GS, de Groot JA, Dutton S (2014). External validation of multivariable prediction models: a systematic review of methodological conduct and reporting. BMC Med Res Methodol.

[40] Collins GS, Ogundimu EO, Altman DG (2016). Sample size considerations for the external validation of a multivariable prognostic model: a resampling study. Stat Med.

[41] Riley RD, Ensor J, Snell KIE (2020). Calculating the sample size required for developing a clinical prediction model. BMJ.

[42] Riley RD, Snell KI, Ensor J (2019). Minimum sample size for developing a multivariable prediction model: PART II —binary and time-to-event outcomes. Stat Med.

[43] Labrique AB, Wadhwani C, Williams KA (2018). Best practices in scaling digital health in low and middle income countries. Global Health.

[44] Ma Y, Zhao C, Zhao Y (2022). Telemedicine application in patients with chronic disease: a systematic review and meta-analysis. BMC Med Inform Decis Mak.

[45] Moons KGM, Altman DG, Reitsma JB (2015). Transparent Reporting of a multivariable prediction model for Individual Prognosis or Diagnosis (TRIPOD): explanation and elaboration. Ann Intern Med.

[46] Snell KIE, Archer L, Ensor J (2021). External validation of clinical prediction models: simulation-based sample size calculations were more reliable than rules-of-thumb. J Clin Epidemiol.

[47] Janson C, Wiklund F, Telg G, Stratelis G, Sandelowsky H (2023). High use of short-acting β_2_-agonists in COPD is associated with an increased risk of exacerbations and mortality. ERJ Open Res.

[48] Rockenschaub P, Hayward A, Shallcross L (2020). Antibiotic prescribing before and after the diagnosis of comorbidity: a cohort study using primary care electronic health records. Clin Infect Dis.

[49] Vollenweider DJ, Frei A, Steurer-Stey CA, Garcia-Aymerich J, Puhan MA (2018). Antibiotics for exacerbations of chronic obstructive pulmonary disease. Cochrane Database Syst Rev.

[50] Benchimol EI, Smeeth L, Guttmann A (2015). The Reporting of studies Conducted using Observational Routinely-collected health Data (RECORD) statement. PLoS Med.

[51] Collins GS, Reitsma JB, Altman DG, Moons KGM (2015). Transparent reporting of a multivariable prediction model for individual prognosis or diagnosis (TRIPOD): the TRIPOD statement. BMJ.

[52] Whittaker H, Rubino A, Müllerová H (2022). Frequency and severity of exacerbations of COPD associated with future risk of exacerbations and mortality: a UK routine health care data study. Int J Chron Obstruct Pulmon Dis.

[53] Bender D, Sartipi K (2013). HL7 FHIR: an agile and RESTful approach to healthcare information exchange.

[54] Jones PW, Harding G, Berry P, Wiklund I, Chen WH, Kline Leidy N (2009). Development and first validation of the COPD Assessment Test. Eur Respir J.

[55] Miravitlles M, Soler-Cataluña JJ, Calle M (2013). A new approach to grading and treating COPD based on clinical phenotypes: summary of the Spanish COPD guidelines (GesEPOC). Prim Care Respir J.

[56] Eklöf J, Sørensen R, Ingebrigtsen TS (2020). Pseudomonas aeruginosa and risk of death and exacerbations in patients with chronic obstructive pulmonary disease: an observational cohort study of 22 053 patients. Clin Microbiol Infect.

[57] Eklöf J, Alispahic IA, Armbruster K (2024). Systemic antibiotics for Pseudomonas aeruginosa infection in outpatients with non-hospitalised exacerbations of pre-existing lung diseases: a randomised clinical trial. Respir Res.

[58] Garcia-Vidal C, Almagro P, Romaní V (2009). Pseudomonas aeruginosa in patients hospitalised for COPD exacerbation: a prospective study. Eur Respir J.

[59] Butler CC, Gillespie D, White P (2019). C-reactive protein testing to guide antibiotic prescribing for COPD exacerbations. N Engl J Med.

[60] Martinez-Garcia MA, Miravitlles M (2022). The impact of chronic bronchial infection in COPD: a proposal for management. Int J Chron Obstruct Pulmon Dis.

[61] Miravitlles M, Anzueto A (2017). Chronic respiratory infection in patients with chronic obstructive pulmonary disease: what is the role of antibiotics?. Int J Mol Sci.

[62] Federman AD, O’Conor R, Nnemnbeng J (2024). Feasibility trial of a comprehensive, highly patient-centered COPD self-management support program. Chronic Obstr Pulm Dis.

[63] Poot CC, Meijer E, Kruis AL, Smidt N, Chavannes NH, Honkoop PJ (2021). Integrated disease management interventions for patients with chronic obstructive pulmonary disease. Cochrane Database Syst Rev.

[64] Bates DW, Saria S, Ohno-Machado L, Shah A, Escobar G (2014). Big data in health care: using analytics to identify and manage high-risk and high-cost patients. Health Aff (Millwood).

[65] Brandsma CA, Van den Berge M, Hackett TL, Brusselle G, Timens W (2020). Recent advances in chronic obstructive pulmonary disease pathogenesis: from disease mechanisms to precision medicine. J Pathol.

[66] Steyerberg EW, Vergouwe Y (2014). Towards better clinical prediction models: seven steps for development and an ABCD for validation. Eur Heart J.

[67] Maltais F, Naya IP, Vogelmeier CF (2020). Salbutamol use in relation to maintenance bronchodilator efficacy in COPD: a prospective subgroup analysis of the EMAX trial. Respir Res.

[68] Fan VS, Gylys-Colwell I, Locke E (2016). Overuse of short-acting beta-agonist bronchodilators in COPD during periods of clinical stability. Respir Med.

[69] Michaux KD, Metcalfe RK, Burns P (2023). Implementing Predictive Analytics towards efficient COPD Treatments (IMPACT): protocol for a stepped-wedge cluster randomized IMPACT study. Diagn Progn Res.

[70] Chen Z, Hao J, Sun H, Li M, Zhang Y, Qian Q (2025). Applications of digital health technologies and artificial intelligence algorithms in COPD: systematic review. BMC Med Inform Decis Mak.

[71] Price D, West D, Brusselle G (2014). Management of COPD in the UK primary-care setting: an analysis of real-life prescribing patterns. Int J Chron Obstruct Pulmon Dis.

[72] Marott JL, Çolak Y, Ingebrigtsen TS, Vestbo J, Nordestgaard BG, Lange P (2022). Exacerbation history, severity of dyspnoea and maintenance treatment predicts risk of future exacerbations in patients with COPD in the general population. Respir Med.

[73] Wynants L, Van Calster B, Collins GS (2020). Prediction models for diagnosis and prognosis of covid-19: systematic review and critical appraisal. BMJ.

[74] Calverley PMA, Anzueto AR, Carter K (2018). Tiotropium and olodaterol in the prevention of chronic obstructive pulmonary disease exacerbations (DYNAGITO): a double-blind, randomised, parallel-group, active-controlled trial. Lancet Respir Med.

[75] Hurst JR, Vestbo J, Anzueto A (2010). Susceptibility to exacerbation in chronic obstructive pulmonary disease. N Engl J Med.

[76] Løkke A, Hilberg O, Lange P, Ibsen R, Bakke P, Ørts LM (2022). The impact on future risk of one moderate COPD exacerbation in GOLD a patients—a cohort study. Eur Respir J.

[77] Miravitlles M, Murio C, Guerrero T, Gisbert R (2003). Costs of chronic bronchitis and COPD: a 1-year follow-up study. Chest.

[78] Viegi G, Pistelli F, Sherrill DL, Maio S, Baldacci S, Carrozzi L (2007). Definition, epidemiology and natural history of COPD. Eur Respir J.

[79] Rajkomar A, Dean J, Kohane I (2019). Machine learning in medicine. N Engl J Med.

[80] Hippisley-Cox J, Coupland C, Vinogradova Y (2008). Predicting cardiovascular risk in England and Wales: prospective derivation and validation of QRISK2. BMJ.

[81] Vayena E, Blasimme A, Cohen IG (2018). Machine learning in medicine: addressing ethical challenges. PLoS Med.

[82] Obermeyer Z, Powers B, Vogeli C, Mullainathan S (2019). Dissecting racial bias in an algorithm used to manage the health of populations. Science.

[83] (2021). Ethics and governance of artificial intelligence for health: WHO guidance. World Health Organization.

[84] Vogelmeier CF, Román-Rodríguez M, Singh D, Han MK, Rodríguez-Roisin R, Ferguson GT (2020). Goals of COPD treatment: focus on symptoms and exacerbations. Respir Med.

