# Supplementary material for: Automated Chronic Obstructive Pulmonary Disease Phenotyping and Control Assessment in Primary Care: Retrospective Multicenter Study Using the Seleida Model
Source: JMIR Med Inform. 2025 Oct 13;13:e74932. doi: 10.2196/74932 (PMC12517459; doi:10.2196/74932)
Supplement: Multimedia Appendix 1 [file medinform-v13-e74932-s001.docx]

**Multimedia Appendix 1.**

**S0. Overview of Supplementary Materials**

This Supplementary Appendix provides the full technical foundation of the Seleida model as implemented and validated in this study. It covers the mathematical formulation, variable definitions, model architecture, comparative validation, and Health Level Seven^®^ (HL7^®^) Fast Healthcare Interoperability Resources (FHIR^®^) interoperability compliance. It is organized into seven sections (S1–S7), each contributing to the model’s transparency, reproducibility, and clinical applicability.

- **S1. Rationale and Functional Justification for Bijectivity in the Seleida Model**

This section explains the functional necessity of demonstrating bijectivity in Seleida. Unlike conventional clinical prediction models that operate in a unidirectional mode (mapping patient data to risk estimates), Seleida is designed for bidirectional inference. This enables both forward computation of risk from clinical data and reverse inference of clinical phenotypes from risk probabilities. To ensure this dual capability, the model must satisfy the mathematical conditions of injectivity (each phenotype yields a unique probability) and surjectivity (each risk value is attainable from at least one phenotype), formally demonstrated in S3.

- **S2. Definition of Core Predictor Variables**

The model relies on two structured, universally available variables from electronic health records (EHRs):

- - *(a)* Annual number of short-acting beta-agonists (SABA) and short-acting muscarinic antagonists (SAMA) canisters dispensed, reflecting reliever use and symptom burden.
  - *(b)* Annual number of antibiotic courses dispensed for bronchitis or chronic obstructive pulmonary disease (COPD) exacerbations, indicating disease instability.

Values for *(a)* and *(b)* were empirically bounded between 0–20 and 0–5, respectively, to preserve the model’s bijective structure and align with real-world clinical distributions.

- **S3. Mathematical Framework and Bijectivity Validation**

This section presents the full logistic regression equation used to compute the probability of poor control (*Pr*), along with its estimated coefficients. Injectivity is demonstrated algebraically, and surjectivity is confirmed computationally through exhaustive mapping of all 126 feasible $(a, b)$ combinations. Uniqueness of output probabilities was validated to 14 decimal places using Python-based verification. This bijective structure underpins Seleida’s reversibility, determinism, and traceability in clinical deployments.

- **S4. Comparative Evaluation of Regularization Strategies**

A head-to-head comparison of four modeling approaches—crude logistic regression, LASSO, Ridge, and Elastic Net—was conducted using 1,000 bootstrap iterations and a standardized evaluation framework. Performance metrics included AUC-ROC, AUC-PR, accuracy, F1-score, log-loss, Brier score, coefficient sparsity, penalization strength (λ), and pseudo-R². LASSO was selected as the optimal method based on its superior balance of interpretability, calibration, and model parsimony. Full results are provided in Supplementary Table S4-1.

- **S5. Exploratory Multivariable Logistic Regression Analysis**

To assess the added value of demographic covariates, we performed an exploratory logistic regression including age group and sex. Although the model showed good overall fit (AUC = 0.971), both variables lacked statistical significance and introduced instability due to sparse subgroup representation. Likelihood ratio tests were non-significant (*P*=.648 for age; *P*=.652 for sex), and estimation artifacts appeared in the Hessian matrix. Detailed diagnostics are in Supplementary Table S5-1. Based on these findings, age and sex were excluded to preserve stability and interpretability.

- **S6. Clinical Interpretation and Use of Seleida Outputs**

This section illustrates how to interpret and apply the two core outputs of the Seleida model: the probability of poor control (*Pr*) and the estimated GOLD ABE phenotype and high or low SABA use (H/L). Through visual examples and clinical decision tables, it demonstrates how Seleida supports both forward risk estimation and reverse phenotyping. These applications guide treatment, inhaled corticosteroid (ICS) optimization, follow-up prioritization, and population-level benchmarking. Figures S6-1 to S6-3 provide practical scenarios and structured guidance for implementation.

- **S7. FHIR Validation and Interoperability Compliance**

To ensure full adherence to international interoperability standards, the Seleida application for automated COPD phenotyping was developed entirely under the HL7^®^ FHIR^®^ R5 specification. All outputs were encoded as structured FHIR resources (Patient, Condition, RiskAssessment, DetectedIssue, Observation, Provenance, Bundle), using standardized attributes and terminology bindings aligned with the FHIR core implementation guide. The final output—one JSON-encoded Batch Bundle per simulated patient—was validated using the official HL7^®^ FHIR^®^ Validator (v6.5.28) against the canonical package hl7.fhir.r5.core#5.0.0, confirming complete syntactic and semantic integrity across 440 resources. SNOMED CT and LOINC terminologies were applied, with permitted extensions introduced where core value sets were insufficient. This validation confirms that the Seleida system enables safe, interoperable, and reproducible deployment within certified EHRs and decision-support environments, ensuring full semantic traceability.

Collectively, these seven sections provide a comprehensive foundation for understanding the Seleida model’s methodological rigor, clinical interpretability, and readiness for real-world translation. From formal proofs of bijectivity to phenotype-driven decision support, and from robust regularization to validated HL7^®^ FHIR^®^ interoperability, each component was designed to ensure reproducibility, safety, and seamless integration into digital health ecosystems. This layered architecture positions Seleida as a scalable, standards-compliant solution for automated COPD stratification. It is compatible with EHR workflows, decision support systems, and federated health data infrastructures.

### **S1. Rationale and Functional Justification for Bijectivity in the Seleida Model**

The Seleida model is built on a bijective logistic framework, meaning that every clinically valid pair of input values—$(a)$ the annual number of SABA/SAMA inhalers dispensed, and $(b)$ the number of antibiotic courses dispensed for respiratory exacerbations—produces one and only one predicted probability of poor COPD control. In turn, each probability value corresponds uniquely to a specific $(a, b)$ combination.

This one-to-one correspondence is not merely a theoretical property; it is essential for Seleida to operate in both directions: predicting risk from clinical data (forward) and identifying the clinical phenotype that underlies a given risk estimate (reverse). Without bijectivity, these operations would be either ambiguous (if different inputs produced the same risk) or incomplete (if some risks had no matching phenotype), making the model unreliable for real-world decision-making.

#### **Mathematical Justification**

The model is defined by a logistic regression equation:

$$logit(Pr) = \alpha+ \beta_{1} \cdot a + \beta_{2} \cdot b ,$$

where *Pr* is the probability of poor control (Y = 1), $\alpha$ is the intercept, and $\beta_{1}$, $\beta_{2}$ are regression coefficients for variables a and b, respectively. The function is bijective within the domain of clinically plausible values for $a\in\mathbb{N}_{a}=\left\{ 0, 1, \ldots, 20 \right\}$ and $b\in\mathbb{N}_{b}=\left\{ 0, 1, \ldots, 5 \right\}.$

Bijectivity requires two conditions:

- **Injectivity**: Each unique input pair $(a, b)$ must produce a distinct and reproducible probability *Pr*. This prevents different phenotypes from being assigned the same risk, which could lead to confusion in clinical decisions.
- **Surjectivity**: Every meaningful probability value between 0 and 1 must be attainable from at least one valid $(a, b)$ combination. This ensures that all thresholds and risk categories are fully defined and clinically usable.

Together, these properties guarantee deterministic, reversible, and fully interpretable behavior—key features that distinguish Seleida from conventional, one-way risk models.

#### **Computational Implications**

Unlike traditional cardiovascular risk scores (e.g., QRISK, Framingham Risk Score), which only infer risk from inputs, Seleida’s bijective mapping enables:

- **Back-projection of thresholds**: determining the minimum $(a, b)$ combination needed to exceed a target risk level (e.g., *Pr*>0.50).
- **Trigger-based classification**: assigning clinical alerts to specific phenotypic patterns for automated decision support.
- **Phenotype-to-risk visualization**: generating interpretable dashboards and heatmaps for implementation and clinical explanation.

If bijectivity were not present, these functions would break down—either mapping multiple phenotypes to the same risk (losing specificity), or leaving gaps in the risk scale (losing completeness). This would compromise transparency, traceability, and reproducibility in digital health environments.

#### **Clinical and Regulatory Relevance**

In real-world primary care—especially in settings without complete spirometry or symptom data—Seleida’s bijective structure ensures that outputs are always safe for deployment in EHR environments. It does so by guaranteeing bidirectional traceability between inputs and outputs, thereby reducing the risk of misclassification and facilitating clinical audits. Specifically, outputs are:

- **Clinically traceable**: Each output can be directly linked to its input values.
- **Reversibly interpretable**: Risk estimates can be translated back into phenotypic patterns for clinical audits and policy rules.
- **Offline-capable**: The model can run in low-resource or disconnected environments without losing interpretability.

In addition, bijectivity supports regulatory compliance by ensuring:

- **Reproducibility**: All results can be replicated using the published equation and coefficients.
- **Deterministic behavior**: A requirement for regulatory approval as a software-as-a-medical-device (SaMD).
- **Third-party validation**: External reviewers can verify model outputs using only the equation and input definitions, without needing access to the training data.

Demonstrating bijectivity is not an abstract mathematical exercise—it is a practical guarantee of how Seleida works. It ensures the model is not only a predictor of COPD control but also a reliable tool for identifying risk-driving phenotypes. This dual capability supports its use in real-time decision support, clinical dashboards, algorithmic audits, and regulatory validation. The formal proof of bijectivity is provided in Section S3.

**S2. Definition of Core Predictor Variables**

The Seleida model uses two standardized input variables, denoted $(a)$ and $(b)$, extracted from structured EHRs. These variables were selected for their high clinical relevance, objectivity, universal documentation across health systems, and strong empirical correlation with poor COPD control [27].

- $\boldsymbol{a}$**:** Annual number of short-acting bronchodilator canisters (SABA and/or SAMA) dispensed per patient, measured in **canisters/year**. This variable serves as a surrogate for symptom burden and reliance on rescue medication.
- $\boldsymbol{b}$**:** Annual number of distinct antibiotic treatment courses prescribed for bronchitis or COPD exacerbations, measured in **courses/year**. This reflects exacerbation frequency and infection-associated instability.

To ensure robustness and maintain the model’s bijective structure, values for:

- $\boldsymbol{a}$ were truncated at 20 canisters/year;
- $\boldsymbol{b}$ were truncated at 5 courses/year.

These thresholds were empirically derived from real-world prescribing distributions to avoid distortion from outliers, saturation effects, or clinically improbable combinations.

The use of these two variables—available in nearly all EHR systems—permits standardized risk estimation across heterogeneous healthcare settings. Their bounded, numeric nature ensures compatibility with embedded decision-support applications, and straightforward interpretation by practitioners, reinforcing the transparency and scalability of the Seleida model.

**S3. Mathematical Framework and Bijectivity Validation**

**Summary of the Bijectivity Proof in the Seleida Model**

To ensure the Seleida model can reliably support both prediction and reverse inference, its **bijective property** was formally demonstrated. This means that:

- Each combination of the two clinical input variables—annual use of SABA/SAMA inhalers and number of antibiotic courses—corresponds to **one and only one** probability of poor COPD control.
- Conversely, **every probability** generated by the model maps uniquely to a single, valid clinical combination.

The proof consists of two components:

1. **Injectivity**: It is shown that no two different input pairs produce the same probability.
2. **Surjectivity**: It is confirmed that all probabilities generated by the model correspond to real clinical input pairs, with no gaps or duplicates.

Both properties were validated using mathematical reasoning and computational verification, confirming that the model is **fully reversible**.

**Practical implications:**

Establishing bijectivity enables the Seleida model to function in **fully automated, remote** settings. Clinical phenotyping of COPD patients can be performed without requiring their physical presence. When the necessary clinical inputs are available, the model can calculate risk and, if needed, reconstruct the underlying clinical profile from the probability alone. This traceability makes Seleida suitable for integration into digital health systems, remote monitoring workflows, and data-driven clinical audits.

**Analytical Demonstration and Empirical Validation of the Seleida Model’s Bijective Mapping**

A rigorous mathematical foundation is essential to ensure the reliability and reproducibility of the Seleida model in clinical practice. This section outlines the key mathematical properties that underpin its predictive and reverse classification capabilities.

The Seleida model’s bijective framework ensures precision and consistency in COPD phenotype classification. Bijectivity establishes a one-to-one correspondence between each probability of poor control and each pair of clinical variables—annual SABA/SAMA canister $(a)$ use and antibiotic courses $(b)$.

Bijective properties (Supplementary Figure 1):

1. **Injectivity:** Each probability maps uniquely to a specific combination of variables, avoiding overlaps in classifications.
2. **Surjectivity:** All clinically relevant variable combinations are represented, ensuring no gaps in phenotype coverage.

These principles enable:

- **Forward mapping:** precise calculation of probabilities from clinical variables.
- **Reverse mapping:** accurate derivation of variable ranges from probabilities, facilitating personalized treatment adjustments.

Monotonic sigmoidal functions ensure that predicted probabilities increase consistently with higher SABA/SAMA use or more frequent antibiotic courses. While monotonicity reinforces interpretability and reliability, it may limit the ability to capture complex non-linear interactions. Embedding bijective principles allows Seleida to move beyond conventional COPD classification, enabling precise phenotyping and real-time, automated treatment optimization.


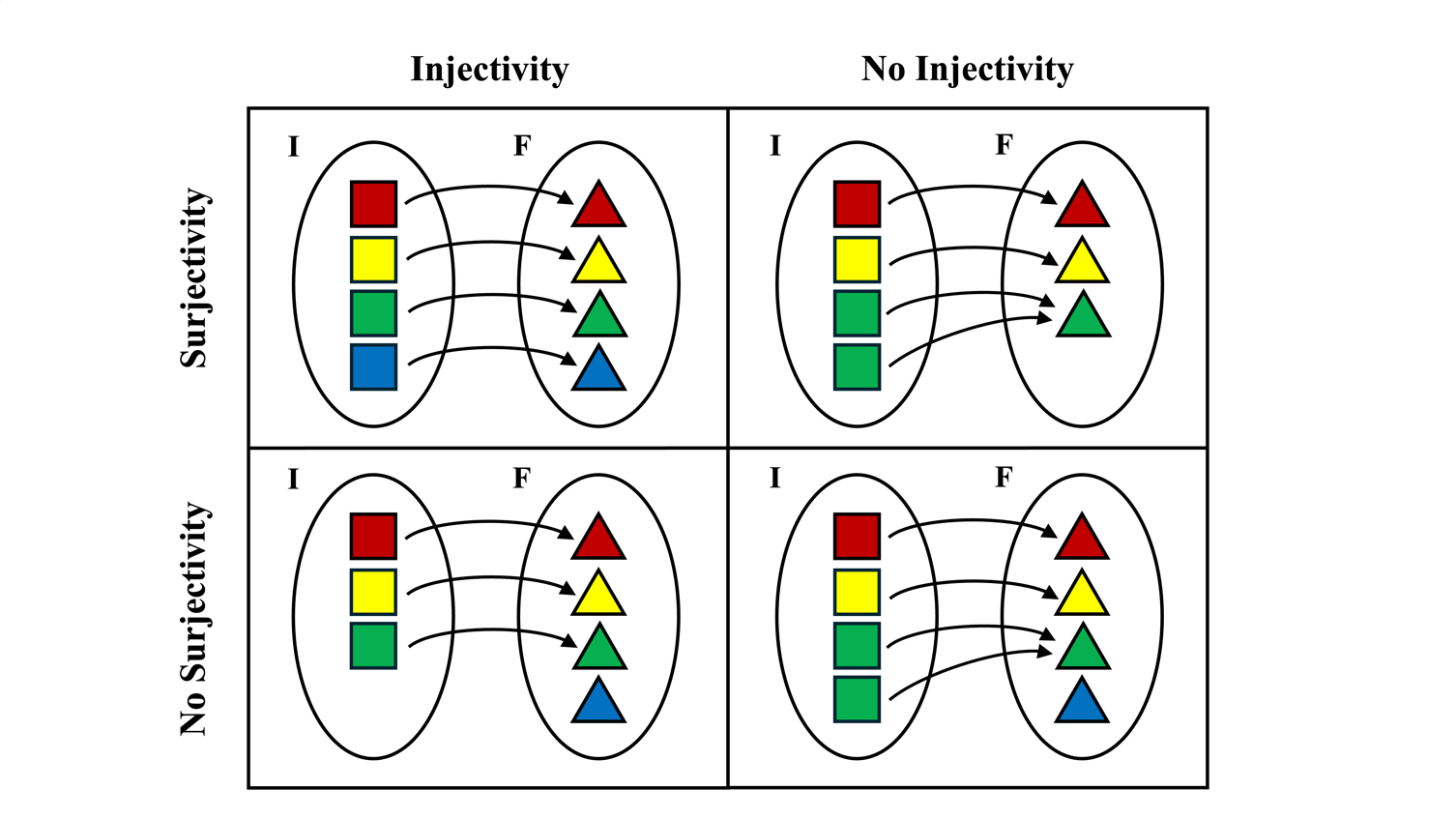


Figure S3-1. Illustration of Injectivity and Surjectivity in Functions. This figure illustrates the relationship between injectivity and surjectivity in mappings from input (I) to output sets (F). The top-left quadrant represents the ideal mapping for the Seleida model, where each input corresponds uniquely to an output, ensuring precise reverse calculations. I, input; F, output.

**Mathematical Proof of Bijectivity**

The mathematical basis of the Seleida model’s bijectivity is demonstrated rigorously as follows:

**Definition**

The probability function of the Seleida model is:

$Pr=\frac{1}{1+e^{-f\left( a,b \right)}}$, $f(a,b)=-1.873+0.427\cdot a+3.654\cdot b$.

Where the bounded values of the natural number variables $a$ and$b$ are:

- $a\in\mathbb{N}_{a}=\left\{ 0, 1, \ldots, 20 \right\}$is the number of annual dispensed SABA/SAMA canisters.
- $b\in\mathbb{N}_{b}=\left\{ 0, 1, \ldots, 5 \right\}$is the number of annual dispensed antibiotic courses.
- $Pr\in(0, 1)$ is the probability of poor control.

***Injectivity***

To demonstrate injectivity, we prove that:$f(a_{1},b_{1}) = f(a_{2}, b_{2}) \Longrightarrow(a_{1}, b_{1}) = (a_{2},b_{2}).$

1. Assume $f(a_{1},b_{1}) = f(a_{2}, b_{2})$.

Then: $-1.873+0.427\cdot a_{1}+3.654\cdot b_{1}=-1.873+0.427\cdot a_{2}+3.654\cdot b_{2}.$

1. Simplify: $0.427\cdot\left( a_{1}- a_{2} \right)+3.654\cdot(b_{1}-b_{2})=0.$
2. As 0.427 and 3.654 are positive constants and $a, b\mathbb{\in N}$, the only solution is: $a_{1}=a_{2}$ and $b_{1}=b_{2}.$

Thus, $f\left( a, b \right)$is injective.

***Surjectivity***

To demonstrate surjectivity, we prove, with a numerical precision of 14 decimals, that for every $Pr\in Im(f)$, there exists a unique pair $(a, b)$ such that:

$Pr=\frac{1}{1+e^{-f\left( a,b \right)}} ,$ $f(a,b)=-1.873+0.427\cdot a+3.654\cdot b$.

The set of probabilities generated by the function is explicitly defined as:

$$Im\left( f \right)=\left\{ Pr\in\left( 0,1 \right) | Pr=\frac{1}{1+e^{-\left( -1.873+0.427\cdot a+3.654\cdot b \right)}}, a\in\mathbb{N}_{a}, b\in\mathbb{N}_{b} \right\}.$$

Here:

• $a\in\mathbb{N}_{a}=\left\{ 0, 1, \ldots, 20 \right\}$is the discrete domain for $a$.

• $b\in\mathbb{N}_{b}=\left\{ 0, 1, \ldots, 5 \right\}$is the discrete domain for $b$.

Explicit computation confirms that each probability $Pr\in Im(f)$corresponds uniquely to a valid pair $(a, b),$ ensuring surjectivity (see Verification of Surjectivity and Bijectivity in the Seleida Model). By proving injectivity and surjectivity, we confirm that the Seleida model possesses the bijective property. As a result, the reverse calculations of this model are mathematically validated, ensuring their accuracy.

**Verification of Surjectivity and Bijectivity in the Seleida Model**

This supplement demonstrates surjectivity, completing the validation of the model’s bijective property. Bijectivity ensures that each probability of poor COPD control ($Pr$) corresponds uniquely to a pair of variables—SABA/SAMA use ($a$) and antibiotic courses ($b$)—and that every possible probability in the model’s discrete theoretical range can be mapped back to a valid $\left( a, b \right)$pair.

Previously, the injective property—ensuring that distinct $(a, b)$ pairs yield distinct probabilities—was demonstrated. To validate surjectivity, we exhaustively computed all possible combinations of $(a,b)$ within their defined bounds $(0 \leq a \leq20; 0 \leq b \leq5)$ and confirmed, at a numerical precision of 14 decimals, that the resulting probability set matches the model’s entire discrete theoretical range, with no gaps or missing values.

Together, these validations confirm the bijectivity of the Seleida model, a property critical for predictive precision and clinical applicability.

**Equation of the Refined Seleida Model**

The probabilities of poor COPD control ($Pr$) are calculated using the following logistic regression equation:

$Pr=\frac{1}{1+e^{-f\left( a,b \right)}}$, $f(a,b)=-1.873+0.427\cdot a+3.654\cdot b$.

Here:

• $a$: annual number of SABA/SAMA canisters ($a\in\mathbb{N}_{a}=\left\{ 0, 1, \ldots, 20 \right\}$).

• $b$: annual number of antibiotic courses ($b\in\mathbb{N}_{b}=\left\{ 0, 1, \ldots, 5 \right\}$).

• $Pr$: probability of poor COPD control ($0<Pr<1$).

**Step 1: Generating the Full Probability Set: the 126-Combination System**

We computed probabilities y for all combinations of $(a, b),$ where a ranges from 0 to 20 and b ranges from 0 to 5, producing a total of 126 combinations. The full list of calculated probabilities is provided below, rounded to 14 decimal places for precision. This precision level exceeds by several orders of magnitude the numerical tolerance of the logistic solver (typically ~1×10⁻⁷), ensuring that any two distinct $(a, b)$ input pairs produce differences in $Pr$ large enough to avoid rounding collisions.

Table S3-1: Predicted probabilities of poor COPD control for each combination of annual rescue inhaler use $(a)$ and antibiotic prescriptions $(b)$, based on the validated Seleida model. All values rounded to 14-decimal precision.

| Antibiotic courses | SABA/SAMA canisters | Poor Control Probability |
| --- | --- | --- |
| $\boldsymbol{b}$ | $\boldsymbol{a}$ | $\boldsymbol{P}$***r*** |
| 0 | 0 | 0.13319497900175 |
| 0 | 1 | 0.19061793336886 |
| 0 | 2 | 0.26522223417531 |
| 0 | 3 | 0.35617609312924 |
| 0 | 4 | 0.45884333184977 |
| 0 | 5 | 0.56512787239084 |
| 0 | 6 | 0.66574443486484 |
| 0 | 7 | 0.75324600394919 |
| 0 | 8 | 0.82390041381905 |
| 0 | 9 | 0.87761111317695 |
| 0 | 10 | 0.91659825225627 |
| 0 | 11 | 0.94395904405055 |
| 0 | 12 | 0.96270902962589 |
| 0 | 13 | 0.97534953163928 |
| 0 | 14 | 0.98377749064648 |
| 0 | 15 | 0.98935539053615 |
| 0 | 16 | 0.99302899182086 |
| 0 | 17 | 0.99544062533767 |
| 0 | 18 | 0.99702045297399 |
| 0 | 19 | 0.99805393901491 |
| 0 | If a≥20 ⇒ a=20 | 0.99872940680018 |
| 1 | 0 | 0.85582030173304 |
| 1 | 1 | 0.90096561672530 |
| 1 | 2 | 0.93308043297193 |
| 1 | 3 | 0.95529778262287 |
| 1 | 4 | 0.97037316024958 |
| 1 | 5 | 0.98046846223996 |
| 1 | 6 | 0.98716928964108 |
| 1 | 7 | 0.99159093193404 |
| 1 | 8 | 0.99449730819502 |
| 1 | 9 | 0.99640281495192 |
| 1 | 10 | 0.99765003004372 |
| 1 | 11 | 0.99846547700945 |
| 1 | 12 | 0.99899824544023 |
| 1 | 13 | 0.99934616403095 |
| 1 | 14 | 0.99957329890099 |
| 1 | 15 | 0.99972155179295 |
| 1 | 16 | 0.99981830511094 |
| 1 | 17 | 0.99988144322092 |
| 1 | 18 | 0.99992264283545 |
| 1 | 19 | 0.99994952591266 |
| 1 | If a≥20 ⇒ a=20 | 0.99996706691998 |
| 2 | 0 | 0.99565770275765 |
| 2 | 1 | 0.99716252726772 |
| 2 | 2 | 0.99814682499919 |
| 2 | 3 | 0.99879009161959 |
| 2 | 4 | 0.99921024679909 |
| 2 | 5 | 0.99948457335302 |
| 2 | 6 | 0.99966364264746 |
| 2 | 7 | 0.99978051342974 |
| 2 | 8 | 0.99985678208810 |
| 2 | 9 | 0.99990655088118 |
| 2 | 10 | 0.99993902587572 |
| 2 | 11 | 0.99996021576322 |
| 2 | 12 | 0.99997404187664 |
| 2 | 13 | 0.99998306311783 |
| 2 | 14 | 0.99998894923665 |
| 2 | 15 | 0.99999278975200 |
| 2 | 16 | 0.99999529556419 |
| 2 | 17 | 0.99999693052208 |
| 2 | 18 | 0.99999799727540 |
| 2 | 19 | 0.99999869329428 |
| 2 | If a≥20 ⇒ a=20 | 0.99999914742176 |
| 3 | 0 | 0.99988711183217 |
| 3 | 1 | 0.99992634169520 |
| 3 | 2 | 0.99995193941050 |
| 3 | 3 | 0.99996864169328 |
| 3 | 4 | 0.99997953962483 |
| 3 | 5 | 0.99998665025601 |
| 3 | 6 | 0.99999128973820 |
| 3 | 7 | 0.99999431685444 |
| 3 | 8 | 0.99999629194734 |
| 3 | 9 | 0.99999758062767 |
| 3 | 10 | 0.99999842144641 |
| 3 | 11 | 0.99999897005075 |
| 3 | 12 | 0.99999932799541 |
| 3 | 13 | 0.99999956154140 |
| 3 | 14 | 0.99999971392170 |
| 3 | 15 | 0.99999981334432 |
| 3 | 16 | 0.99999987821396 |
| 3 | 17 | 0.99999992053905 |
| 3 | 18 | 0.99999994815462 |
| 3 | 19 | 0.99999996617278 |
| 3 | If a≥20 ⇒ a=20 | 0.99999997792897 |
| 4 | 0 | 0.99999707730062 |
| 4 | 1 | 0.99999809304324 |
| 4 | 2 | 0.99999875577940 |
| 4 | 3 | 0.99999918819105 |
| 4 | 4 | 0.99999947032410 |
| 4 | 5 | 0.99999965440572 |
| 4 | 6 | 0.99999977451230 |
| 4 | 7 | 0.99999985287748 |
| 4 | 8 | 0.99999990400792 |
| 4 | 9 | 0.99999993736866 |
| 4 | 10 | 0.99999995913533 |
| 4 | 11 | 0.99999997333729 |
| 4 | 12 | 0.99999998260356 |
| 4 | 13 | 0.99999998864945 |
| 4 | 14 | 0.99999999259418 |
| 4 | 15 | 0.99999999516797 |
| 4 | 16 | 0.99999999684728 |
| 4 | 17 | 0.99999999794297 |
| 4 | 18 | 0.99999999865786 |
| 4 | 19 | 0.99999999912430 |
| 4 | If a≥20 ⇒ a=20 | 0.99999999942864 |
| If b≥5 ⇒ b=5 | 0 | 0.99999992433878 |
| If b≥5 ⇒ b=5 | 1 | 0.99999995063381 |
| If b≥5 ⇒ b=5 | 2 | 0.99999996779036 |
| If b≥5 ⇒ b=5 | 3 | 0.99999997898438 |
| If b≥5 ⇒ b=5 | 4 | 0.99999998628808 |
| If b≥5 ⇒ b=5 | 5 | 0.99999999105347 |
| If b≥5 ⇒ b=5 | 6 | 0.99999999416272 |
| If b≥5 ⇒ b=5 | 7 | 0.99999999619138 |
| If b≥5 ⇒ b=5 | 8 | 0.99999999751502 |
| If b≥5 ⇒ b=5 | 9 | 0.99999999837864 |
| If b≥5 ⇒ b=5 | 10 | 0.99999999894212 |
| If b≥5 ⇒ b=5 | 11 | 0.99999999930977 |
| If b≥5 ⇒ b=5 | 12 | 0.99999999954965 |
| If b≥5 ⇒ b=5 | 13 | 0.99999999970616 |
| If b≥5 ⇒ b=5 | 14 | 0.99999999980828 |
| If b≥5 ⇒ b=5 | 15 | 0.99999999987491 |
| If b≥5 ⇒ b=5 | 16 | 0.99999999991838 |
| If b≥5 ⇒ b=5 | 17 | 0.99999999994675 |
| If b≥5 ⇒ b=5 | 18 | 0.99999999996526 |
| If b≥5 ⇒ b=5 | 19 | 0.99999999997733 |
| If b≥5 ⇒ b=5 | If a≥20 ⇒ a=20 | 0.99999999998521 |

**Step 2: Validating Surjectivity**

To verify surjectivity, we ensured that every calculated probability $Pr$ in the table corresponds uniquely to one specific $(a, b)$ pair. This was accomplished programmatically using Python in a Google Colab™ environment.

**Python Code for Validation**

The following Python script iterates over the computed probabilities, checks for duplicates, and confirms whether all probabilities ($Pr$) are unique.

def check_unique_probabilities(probabilities):

"""

Function to check if all probabilities in the list are unique.

:param probabilities: List of probabilities to check.

:return: None. Prints a confirmation message.

"""

unique_probs = set() # Set to store unique probabilities

for prob in probabilities:

if prob in unique_probs:

print(f"Duplicate probability found: {prob}")

return # Exit if a duplicate is found

unique_probs.add(prob)

print("All probabilities are unique. No duplicates found.")

# List of probabilities with 14 decimal places

probabilities = [

0.13319497900175, 0.19061793336886, 0.26522223417531, 0.35617609312924,

0.45884333184977, 0.56512787239084, 0.66574443486484, 0.75324600394919,

0.82390041381905, 0.87761111317695, 0.91659825225627, 0.94395904405055,

0.96270902962589, 0.97534953163928, 0.98377749064648, 0.98935539053615,

0.99302899182086, 0.99544062533767, 0.99702045297399, 0.99805393901491,

0.99872940680018, 0.85582030173304, 0.90096561672530, 0.93308043297193,

0.95529778262287, 0.97037316024958, 0.98046846223996, 0.98716928964108,

0.99159093193404, 0.99449730819502, 0.99640281495192, 0.99765003004372,

0.99846547700945, 0.99899824544023, 0.99934616403095, 0.99957329890099,

0.99972155179295, 0.99981830511094, 0.99988144322092, 0.99992264283545,

0.99994952591266, 0.99996706691998, 0.99565770275765, 0.99716252726772,

0.99814682499919, 0.99879009161959, 0.99921024679909, 0.99948457335302,

0.99966364264746, 0.99978051342974, 0.99985678208810, 0.99990655088118,

0.99993902587572, 0.99996021576322, 0.99997404187664, 0.99998306311783,

0.99998894923665, 0.99999278975200, 0.99999529556419, 0.99999693052208,

0.99999799727540, 0.99999869329428, 0.99999914742176, 0.99988711183217,

0.99992634169520, 0.99995193941050, 0.99996864169328, 0.99997953962483,

0.99998665025601, 0.99999128973820, 0.99999431685444, 0.99999629194734,

0.99999758062767, 0.99999842144641, 0.99999897005075, 0.99999932799541,

0.99999956154140, 0.99999971392170, 0.99999981334432, 0.99999987821396,

0.99999992053905, 0.99999994815462, 0.99999996617278, 0.99999997792897,

0.99999707730062, 0.99999809304324, 0.99999875577940, 0.99999918819105,

0.99999947032410, 0.99999965440572, 0.99999977451230, 0.99999985287748,

0.99999990400792, 0.99999993736866, 0.99999995913533, 0.99999997333729,

0.99999998260356, 0.99999998864945, 0.99999999259418, 0.99999999516797,

0.99999999684728, 0.99999999794297, 0.99999999865786, 0.99999999912430,

0.99999999942864, 0.99999992433878, 0.99999995063381, 0.99999996779036,

0.99999997898438, 0.99999998628808, 0.99999999105347, 0.99999999416272,

0.99999999619138, 0.99999999751502, 0.99999999837864, 0.99999999894212,

0.99999999930977, 0.99999999954965, 0.99999999970616, 0.99999999980828,

0.99999999987491, 0.99999999991838, 0.99999999994675, 0.99999999996526,

0.99999999997733, 0.99999999998521

]

# Verify uniqueness of probabilities

if len(probabilities) == len(set(probabilities)):

print("All probabilities are unique. No duplicates found.")

else:

print("Duplicate probabilities detected!")

**Validation Results**


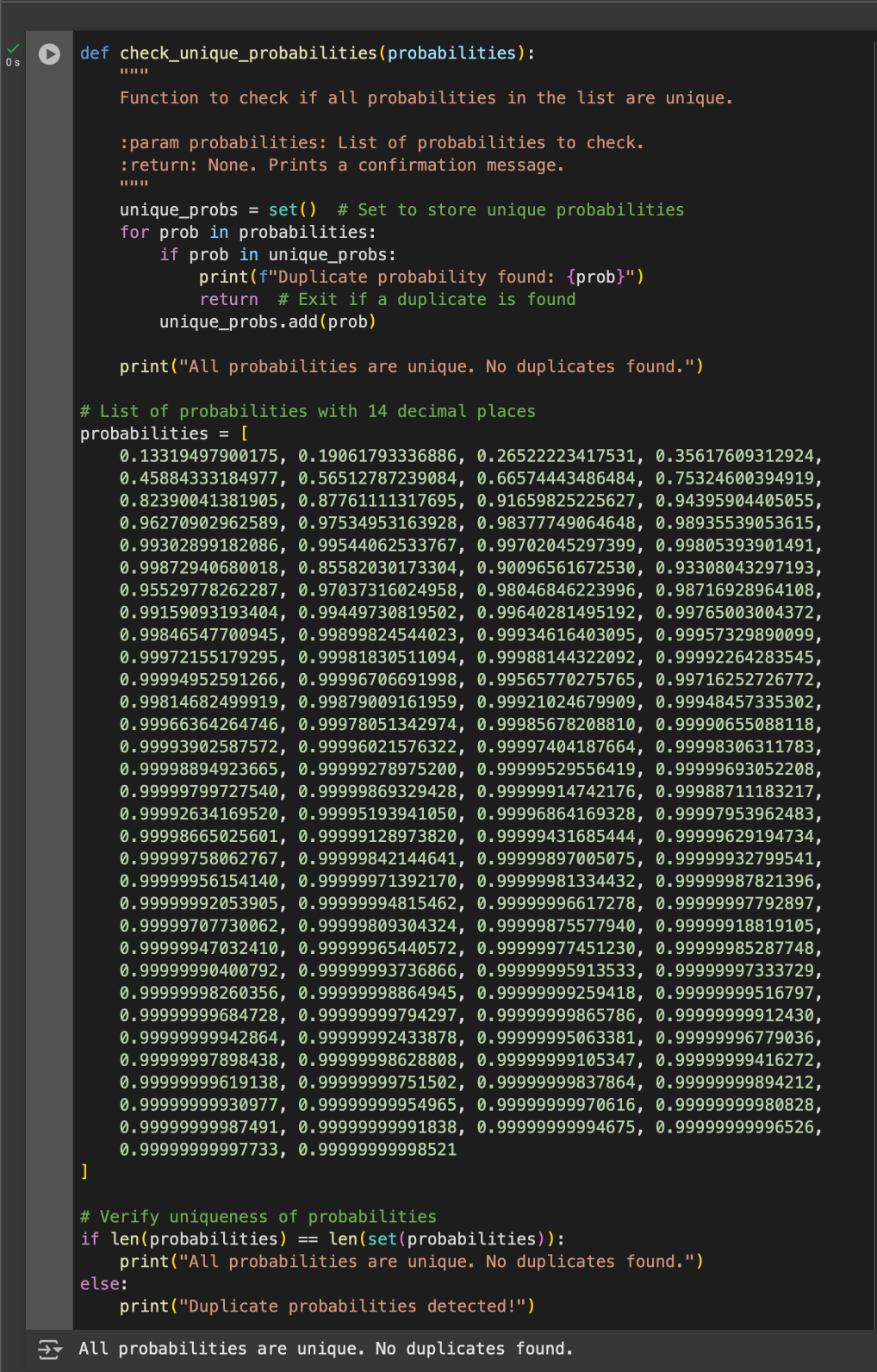


*Python validation script output confirming the uniqueness of probabilities (surjectivity verification) in the Seleida model. Capture generated on 2025-01-06. 19:59:53 h.*

Upon executing the script, the following output was generated:

**“All probabilities are unique. No duplicates found.”**

This result confirms that no duplicate probabilities exist within the calculated set. thereby satisfying the criterion for **surjectivity**.

**Step 3: Demonstrating Bijectivity**

The successful validation of surjectivity, combined with the previously established injectivity—proving that each *(a, b)* pair maps to a unique $Pr$—, confirms the Seleida model’s bijective property. This guarantees:

1. Each $(a, b)$ maps uniquely to a distinct $Pr$.
2. Every valid probability $Pr$ within the model’s range corresponds to exactly one pair $(a, b)$.

**Conclusion**

The Seleida model’s bijective structure—proven analytically and validated computationally—ensures a one-to-one correspondence between clinical input pairs ($a, b$) and the probability of poor COPD control ($Pr$). This property enables both accurate forward prediction and exact reverse inference, supporting phenotyping, control assessment, and traceable integration into clinical and informatics systems.

By preserving the original clinical information in Pr, bijectivity guarantees that reverse mapping remains valid, interpretable, and reproducible—supporting transparent clinical decisions and system-level audits. Through rigorous proof and high-precision verification, Seleida becomes a deterministic, reversible classifier suited for automated COPD phenotyping, real-time stratification, and scalable deployment.

**S4. Comparative Evaluation of Regularization Strategies**

To empirically determine the most appropriate regularization method for the Seleida model, we compared four logistic regression approaches: the unpenalized reference model, Least Absolute Shrinkage and Selection Operator (LASSO, L1 regularization), Ridge (L2 regularization), and Elastic Net (combined L1 + L2). All models used the same binary outcome—poor vs. good COPD control—and the same predictors: annual dispensations of SABA/SAMA and respiratory antibiotics. No variable scaling was applied, preserving the clinical interpretability of coefficients. Internal validation used 1,000 bootstrap iterations, and λ (penalty parameter) values were optimized via five-fold cross-validation using the minimum deviance criterion. Performance metrics and 95% confidence intervals (CI) were derived from the bootstrap distributions.

Table S4-1. Comparative Performance of Logistic Models With and Without Regularization. AUC-ROC, area under the receiver operating characteristic curve; AUC-PR, area under the precision–recall curve; PPV, positive predictive value; NPV, negative predictive value; F1-score, harmonic mean of precision and recall; Brier, Brier score (mean squared error of predicted probabilities); Log Loss, negative log-likelihood; Lambda (λ), regularization penalty parameter selected by cross-validation; McFadden’s R², pseudo-R-squared; Coefficient magnitude, average absolute value of non-zero coefficients; CI, confidence interval estimated via bootstrap (n = 1,000); SABA, short-acting beta-agonists; SAMA, short-acting muscarinic antagonists.

| Metric | Unpenalized | LASSO | Ridge | ElasticNet |
| --- | --- | --- | --- | --- |
| Intercept [95% CI] | -2.548 [-4.033–-1.770] | -2.313 [-3.656–-1.615] | -1.193 [-1.576–-0.848] | -2.348 [-3.663–-1.685] |
| SABA/SAMA [95% CI] | 0.454 [0.230–1.304] | 0.422 [0.215–1.187] | 0.356 [0.195–0.703] | 0.426 [0.227–1.208] |
| Antibiotic courses [95% CI] | 5.231 [3.458–22.685] | 4.181 [2.831–9.865] | 1.061 [0.736–1.483] | 4.275 [2.863–8.029] |
| AUC-ROC | 0.9747 | 0.9732 | 0.9495 | 0.9711 |
| AUC-PR | 0.9789 | 0.9776 | 0.9441 | 0.9751 |
| Accuracy | 0.8962 | 0.8962 | 0.8585 | 0.8962 |
| Sensitivity | 0.8621 | 0.8621 | 0.8276 | 0.8621 |
| Specificity | 0.9375 | 0.9375 | 0.8958 | 0.9375 |
| PPV | 0.9434 | 0.9434 | 0.9057 | 0.9434 |
| NPV | 0.8491 | 0.8491 | 0.8113 | 0.8491 |
| F1-score | 0.9009 | 0.9009 | 0.8649 | 0.9009 |
| Brier Score | 0.0741 | 0.0740 | 0.1131 | 0.0739 |
| Log Loss | 0.2451 | 0.2490 | 0.3831 | 0.2491 |
| Lambda (λ , penalty) | NA | 0.0093 | 0.0973 | 0.0038 |
| McFadden’s R^2^ | 0.6442 | 0.3616 | 0.5563 | 0.3617 |
| Variables retained | 3 | 2 | 2 | 2 |
| Coefficient Magnitude | 2.6893 | 2.2163 | 0.7064 | 2.2141 |

**Coefficient Discrepancies and Methodological Justification**

The coefficient values reported differ slightly from those published in the initial Seleida development study. These differences are expected and justifiable, arising mainly from the analytical frameworks used. The original estimates were generated in R (version 4.3.4.2) using *glmnet*, whereas this analysis was performed in Python 3.11 using *scikit-learn* and *statsmodels*. These platforms differ in numerical solvers, convergence criteria, and penalization paths, which can lead to minor coefficient variations even with identical datasets and model structures.

Additionally, this analysis incorporates 1,000 bootstrap iterations to produce empirical CIs, reflecting the sampling variability of real-world data. This resampling—absent from the original study—enhances robustness but introduces small variations in point estimates. Importantly, all original coefficients fall within the 95% CIs obtained here, confirming full compatibility. These differences reflect methodological variation rather than model instability, supporting reproducibility across environments.

### **Discrimination and Calibration**

The unpenalized model achieved the highest discrimination (AUC-ROC = 0.9747; AUC-PR = 0.9789). LASSO and Elastic Net showed minimal degradation (ΔAUC < 0.004), whereas Ridge underperformed (AUC-ROC = 0.9495), with corresponding declines in AUC-PR and calibration metrics. Log-loss and Brier scores indicated that LASSO and Elastic Net preserved predictive accuracy and probability calibration more effectively than Ridge.

### **Coefficient Behavior and Model Parsimony**

LASSO and Elastic Net retained both predictors (SABA/SAMA and antibiotic courses) with effect sizes close to the unpenalized model, supporting interpretability. Ridge excessively shrank the antibiotic coefficient (1.061 vs. 5.231), potentially reducing clinical relevance. LASSO and Elastic Net achieved a moderate reduction in the absolute coefficient values without loss of discrimination, thus improving parsimony.

### **Penalization Strength and Overall Fit**

Cross-validated λ values confirmed theoretical expectations: Ridge applied stronger penalization (λ = 0.0973), followed by LASSO (λ = 0.0093) and Elastic Net (λ = 0.0038). The lower McFadden’s R² observed in penalized models reflects the shrinkage of likelihood space, not inferior performance per se, as evidenced by stable discrimination and calibration metrics across models.

### **Justification for Regularization Method**

LASSO was selected as the preferred regularization strategy because it:

- Preserved the magnitude, direction, and statistical relevance of both predictors.
- Produced performance metrics nearly identical to the full model.
- Achieved variable selection without degrading model accuracy.
- Avoided Ridge’s excessive shrinkage and Elastic Net’s added complexity.

In low-dimensional settings with minimal multicollinearity, LASSO offers the optimal balance between interpretability, robustness, and generalizability. Its embedded feature selection and minimal computational cost support deployment in resource-limited or real-time clinical environments.

This comparative analysis shows that LASSO regularization preserves the predictive accuracy of the unpenalized logistic model while improving parsimony, coefficient stability, and operational simplicity. These properties make LASSO the most appropriate penalization method for the Seleida model and reinforce its suitability for transparent, scalable, and clinically interpretable implementation.

## **S5. Exploratory Multivariable Logistic Regression Analysis**

To evaluate whether adding demographic variables could improve the predictive performance of the Seleida model, we conducted an exploratory multivariable logistic regression incorporating age group and sex as additional covariates. The binary outcome was COPD control status (well-controlled vs. poorly controlled), defined according to predefined clinical thresholds. The independent variables were:

- Age group (40–49, 50–59, 60–69, 70–79, and 80 years).
- Sex (male, female).
- Annual number of SABA/SAMA canisters dispensed per patient.
- Annual number of antibiotic dispensations issued for bronchitis or COPD exacerbations.

#### Table S5-1. Predictors of Poor COPD Control in the Multivariable Logistic Regression Model (n = 106). COPD, chronic obstructive pulmonary disease.

| Predictor | Coefficient (β) | OR | 95% CI (OR) | *P*-value |
| --- | --- | --- | --- | --- |
| (Intercept) | 14.460 | — | — | .9952 |
| Sex (male vs. female) | 0.434 | 1.544 | 0.283 – 8.417 | .6157 |
| Age: 50–60 years | −16.880 | 0.000 | 0.000 – ∞ | .9944 |
| Age: 60–70 years | −17.350 | 0.000 | 0.000 – ∞ | .9942 |
| Age: 70–80 years | −17.001 | 0.000 | 0.000 – ∞ | .9943 |
| Age = 80 years | −17.483 | 0.000 | 0.000 – ∞ | .9942 |
| SABA/SAMA canisters | 0.421 | 1.524 | 1.186 – 1.958 | .0010^a^ |
| Antibiotic courses | 5.060 | 157.5 | 16.675 – 1488.189 | <.0001 ^b^ |

^a^ *P<.01,* ^b^*P<.001.*

#### **Model Fit and Discrimination**

The expanded model showed strong overall performance (−2 log-likelihood = 30.10; model χ² = 110.93, df = 23, *P*<.001). Pseudo-R² values indicated high explanatory power (Nagelkerke = 0.868; McFadden = 0.760; Cox & Snell = 0.649). The area under the receiver operating characteristic curve (AUC-ROC) was 0.971, reflecting excellent discrimination. However, this level of performance was almost entirely driven by the two primary predictors—annual SABA/SAMA dispensations and annual respiratory antibiotic courses—rather than by the demographic covariates.

#### **Predictor Significance and Model Stability**

Both SABA/SAMA use and antibiotic courses remained highly significant (*P*= .0010 and *P*<.0001), consistent with their role in the primary Seleida model. In contrast, age group (Wald χ² = 2.48, df = 4, *P*=.648) and sex (Wald χ² = 0.203, df = 1, *P*=.652) showed no independent predictive value. Coefficients for age strata displayed instability, with inflated standard errors, extreme odds ratios (e.g., OR > 10⁸), and convergence warnings—indicative of quasi-complete separation from sparse observations in certain subgroups (e.g., 80 years without exacerbations).

#### **Justification for Model Simplification**

Although global fit metrics appeared favorable, adding age and sex provided no meaningful predictive gain and introduced estimation noise and numerical instability. Exclusion of these covariates was based on four criteria:

1. Lack of statistical significance and absence of incremental explanatory value.
2. Estimation instability, including collinearity and separation artifacts.
3. Violation of parsimony principle—critical in small-sample predictive modeling
4. Alignment with the primary binary model, in which the same two predictors achieved equal or better discrimination with greater robustness.

These findings reinforce the minimalist design of the Seleida model, which focuses on generalizability, operational feasibility in real-world settings, and interpretability across diverse healthcare environments. By retaining only the two core predictors—SABA/SAMA use and antibiotic courses—the model avoids overfitting, maximizes robustness, and preserves its ability to function in settings with limited demographic data availability.

**S6. Clinical Interpretation and Use of Seleida Outputs**

To facilitate real-world implementation, this section illustrates how to interpret and apply the two core outputs of the Seleida model:

- the **probability of poor control (*Pr*)**, and
- the **estimated GOLD ABE phenotype**, derived from structured EHR inputs.

These outputs are intended for direct use in both individual consultations and population-level workflows, supporting treatment adjustment, inhaled corticosteroid (ICS) optimization, microbial risk detection, and epidemiological surveillance.


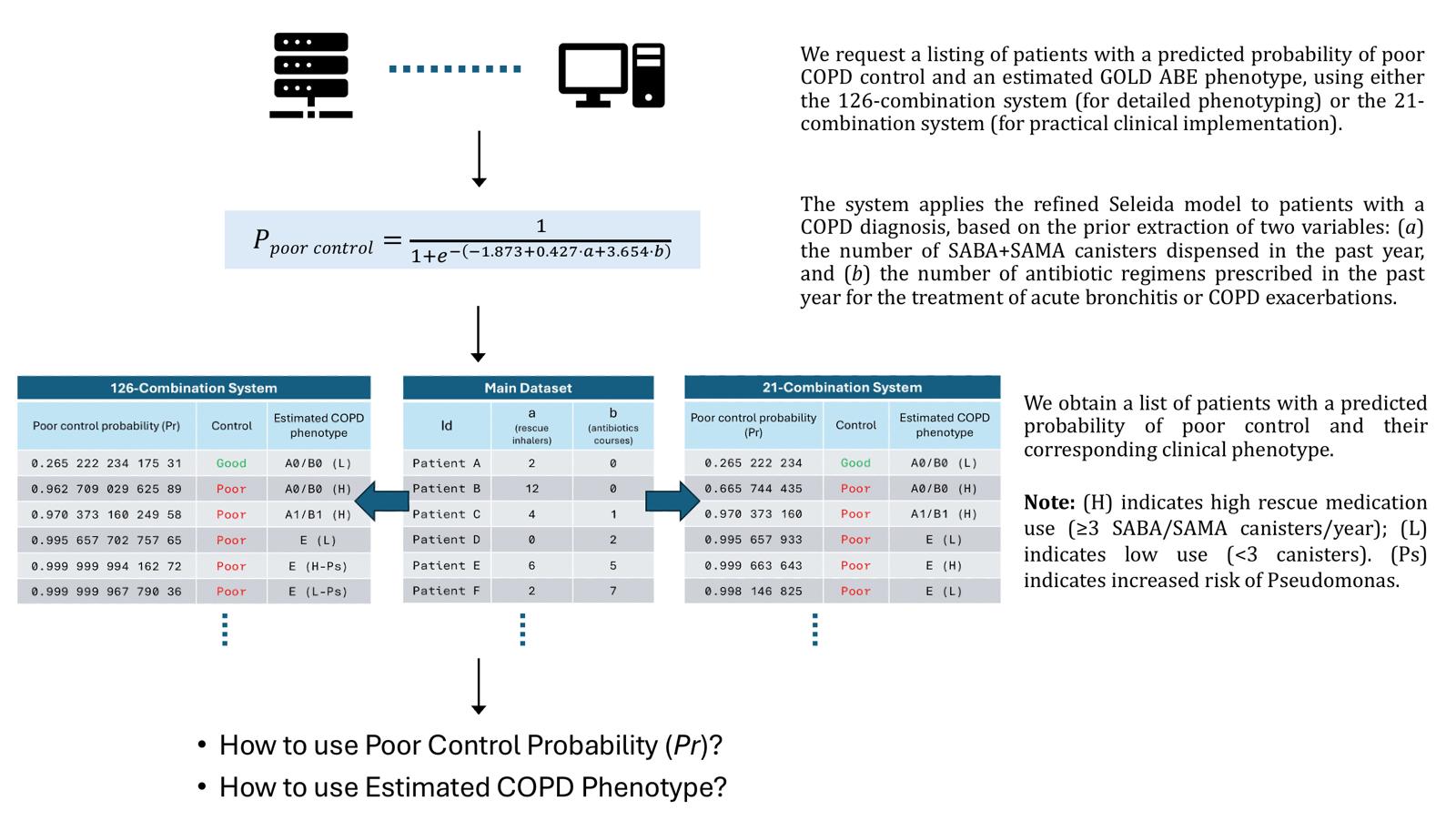


Figure S6-1 presents an example of how patient-level data (*a* = rescue inhalers, *b* = antibiotic courses) are processed through the Seleida model to generate individualized outputs.


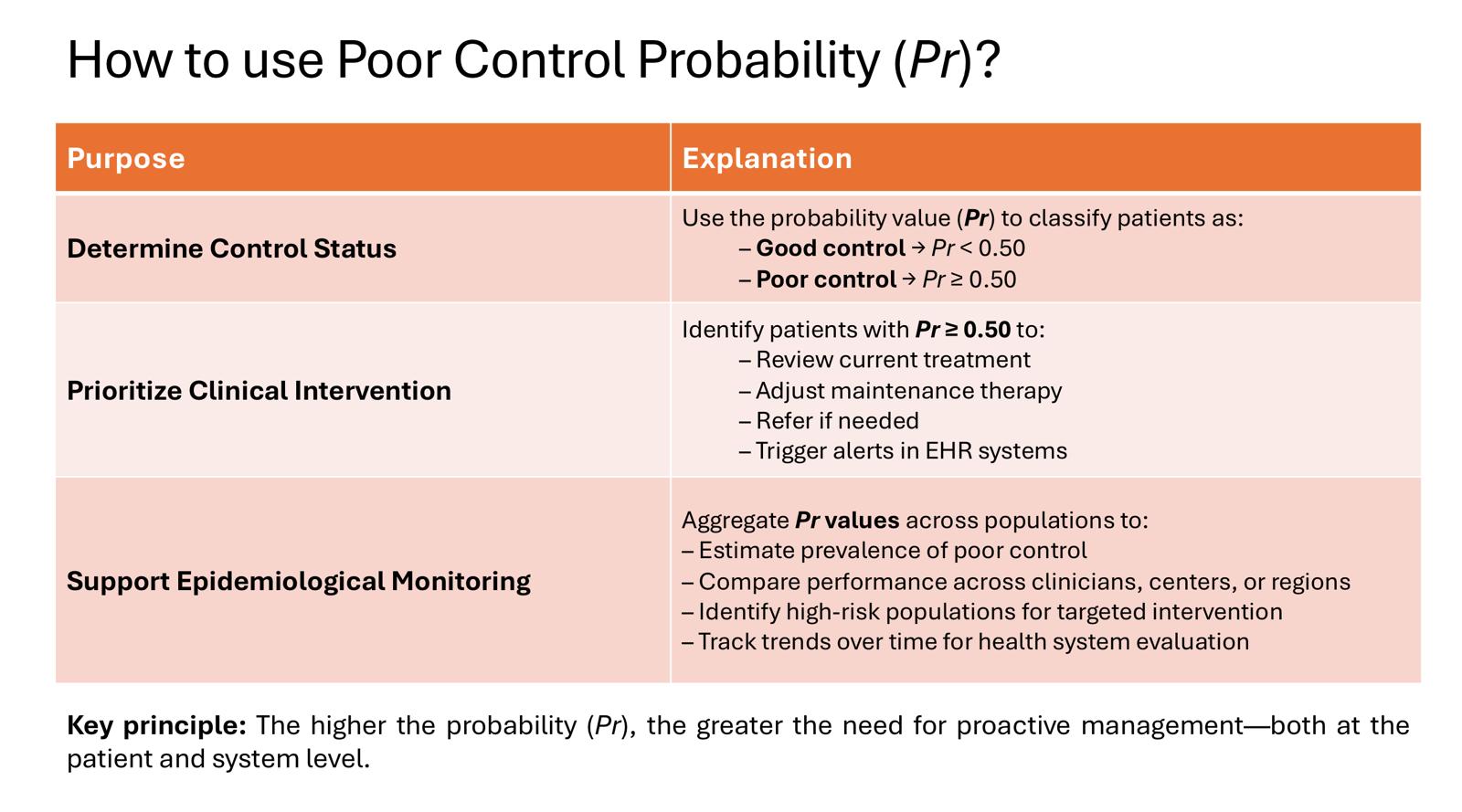
Figure S6-2 summarizes how to interpret and act upon the predicted probability (*Pr*), both at the patient level and across clinical systems. EHR, electronic health record.


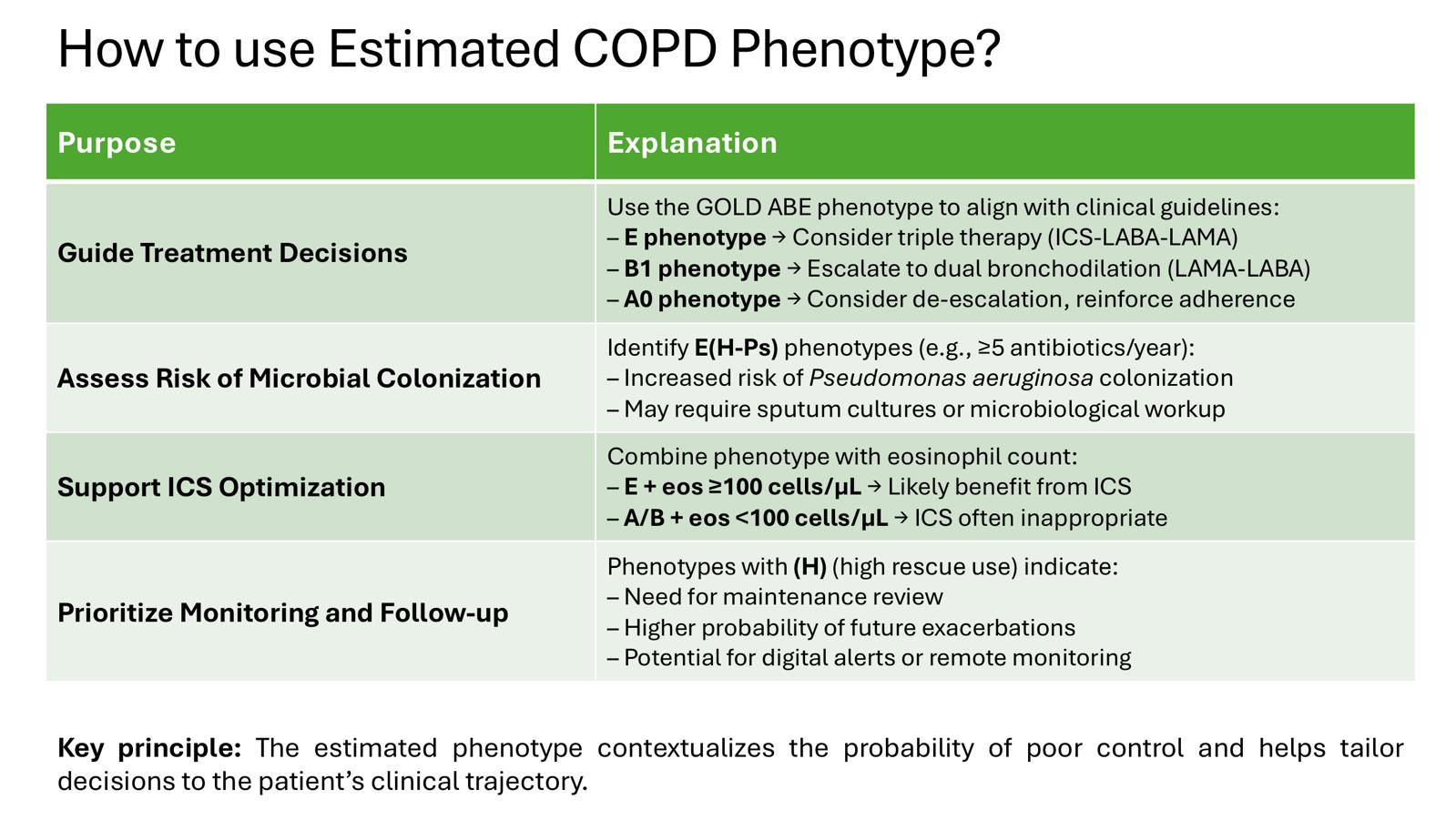


Figure S6-3 outlines how the estimated COPD phenotype can guide clinical decisions, including therapeutic escalation, de-escalation, follow-up intensity, and microbiological investigation when needed. Abbreviations: ABE: GOLD 2025 classification (A0 = no exacerbations, B1 = one moderate, E = ≥2 moderate or ≥1 severe), (H): High rescue inhaler use (≥3 SABA/SAMA canisters/year), (L): Low rescue inhaler use (<3 canisters/year), (Ps): High risk of *Pseudomonas aeruginosa* colonization (typically b ≥ 5), ICS: Inhaled corticosteroids, LABA: Long-acting β₂-agonist, LAMA: Long-acting muscarinic antagonist, and eos: Blood eosinophil count (cells/μL).

Together, these tools convert routine prescribing patterns into actionable insights, enabling precision care and scalable monitoring without requiring spirometry or symptom scores.

**S6.1. Implementation in Primary Care: A Family Physician’s Perspective**

The Seleida model generates two core outputs for patients with COPD—namely, the estimated probability of poor control (*Pr*) and the refined GOLD ABE phenotype—based on real-world prescribing data. These outputs enable scalable identification of clinically unstable patients, without requiring spirometry or symptom scores, and are directly actionable in primary care settings.

To translate these outputs into clinical decisions, a structured management pathway can be established that integrates model-driven stratification with guideline-recommended interventions. This section describes how family physicians can prioritize and intervene on patients with elevated P values, using the GOLD 2025 framework and real-world risk indicators inferred from medication patterns.

#### **Step 1: Risk-Based Prioritization**

Patients are ranked by *Pr*, with higher values indicating greater risk of exacerbations, hospitalization, or silent disease progression. Table S6-1 presents six illustrative patients (A–F) ordered by ascending *Pr*, along with their GOLD ABE phenotype and relevant risk modifiers—such as high rescue inhaler use (H) and frequent antibiotics (Ps) linked to potential *Pseudomonas aeruginosa* colonization.

Table S6-1. Seleida-based stratification of six illustrative COPD patients (A–F), ordered by predicted probability of poor control (*Pr*). Each patient is classified by their corresponding GOLD ABE phenotype and associated risk modifiers. High rescue inhaler use is denoted as (H), low use as (L), and frequent antibiotic prescriptions (≥5/year) as (Ps), indicating potential *Pseudomonas aeruginosa* colonization. This stratification supports clinical prioritization and targeted intervention.

| Rank | Patient | *Pr* Value | Seleida Phenotype | Clinical Risk Level |
| --- | --- | --- | --- | --- |
| 1 | E | 0.999 | E (H-Ps) | Very high (symptomatic + infection risk) |
| 2 | F | 0.999 | E (L-Ps) | Very high (silent + infection risk) |
| 3 | D | 0.995 | E (L) | High (underperceived symptoms) |
| 4 | C | 0.973 | A1/B1 (H) | Moderate (symptomatic, no exacerbations) |
| 5 | B | 0.665 | A0/B0 (H) | Moderate (high use, no exacerbations) |
| 6 | A | 0.265 | A0/B0 (L) | Low (stable profile) |

#### **Step 2: Structured Clinical Review**

For all patients with ***Pr*≥0.50**, a structured clinical reassessment should be initiated to identify potentially modifiable contributors to poor control, and GOLD 2025 recommends an integrated review that includes:

- **Inhaler technique** (including device resistance and inspiratory flow adequacy).
- **Therapeutic adherence**, using pharmacy refill data or direct questioning.
- **Pharmacological appropriateness**: verifying correct drug class, molecule, dose, and delivery system.
- **Identification of unrecognized comorbidities and management of those that are uncontrolled** (e.g., cardiovascular disease, anxiety, gastroesophageal reflux disease).
- **Assessment of reliever overuse** as a surrogate for symptom burden or undertreatment.

Patients such as C and B, who demonstrate elevated *Pr* with high reliever use but no documented exacerbations, may reflect under-treated dyspnea, inappropriate maintenance regimens, or suboptimal inhaler-device matching. In contrast, patients E and F—with frequent antibiotic use (≥5 courses/year)—warrant a microbiological work-up to evaluate chronic Pseudomonas colonization, consideration of triple inhaled therapy according to GOLD criteria, sputum culture, and, where appropriate, referral to a hospital-based specialist.

**Step 3: Tailored Therapeutic Adjustments**

Following clinical reassessment, therapeutic interventions should align with both the predicted control status (*Pr*) and the estimated GOLD ABE phenotype:

- **E phenotype** → Strong indication for escalation to triple inhaled therapy (LABA+LAMA+ICS), particularly in patients with blood eosinophils ≥300 cells/µL.
- **A0/B1 phenotypes** → Candidates for dual bronchodilation (LABA+LAMA) with ICS de-escalation in low-eosinophil profiles (<100 cells/µL).
- **EH-Ps subtypes** → Require microbiological surveillance and sputum culture due to increased risk of resistant pathogens (e.g., *P. aeruginosa*).
- **High-rescue users** (H) → Trigger alerts for medication review and behavioral interventions (e.g., technique re-training, adherence counseling).

Patients with discordant patterns (e.g., high *Pr* + low rescue use, as seen in F) should not be overlooked, as they may represent “silent progressors” with poor symptom perception or unrecognized frequent exacerbations. In such cases, structured follow-up (in-person or remote) and close monitoring are essential.

#### **Step 4: Follow-up**

Seleida outputs can be revisited longitudinally to evaluate therapeutic response and control status evolution. Reclassification of patients should be performed after each treatment adjustment, using updated prescription data, to ensure dynamic alignment between predicted risk and real-world management.

### **Conclusion**

Combining *Pr*-based stratification with phenotype-guided interventions helps family physicians close therapeutic gaps, reduce exacerbation risk, and individualize COPD care—without reliance on spirometry or symptom scores.

**S6.2. Population-Level Audit and Infection Risk Surveillance: A Health Management Perspective**

The application of the Seleida model extends beyond individual clinical management to support data-driven population health strategies. From the standpoint of a healthcare directorate, hospital pharmacy, or epidemiology service, Seleida enables real-time monitoring of COPD treatment adequacy, audit of pharmacological consistency with current guidelines, and early detection of patients at microbiological risk.

This section outlines how Seleida can be deployed as a surveillance tool to systematically identify:

(1) patients with poorly controlled COPD who are receiving suboptimal therapy,

(2) overtreatment or ICS misuse among well-controlled individuals, and

(3) patients at high risk of *Pseudomonas aeruginosa* colonization eligible for microbiological work-up and referral to hospital-based specialist care.

**Step 1. Treatment Appropriateness Audit**

Seleida-generated probability scores (*Pr*) allow COPD patients to be stratified into clinically stable (*Pr*<0.50) and unstable (*Pr*≥0.50) groups. Once stratified, their pharmacological regimens can be benchmarked against GOLD 2025 guidelines to determine alignment between therapeutic intensity and actual risk status.

A standardized audit matrix can be applied (Table S6-2).

Table S6-2. Alignment between Seleida-predicted control status and guideline-based pharmacological strategies. Patients with a high probability of poor control (*Pr*≥0.50) are expected to receive escalation to dual or triple therapy depending on blood eosinophil count. Conversely, those with low *Pr* values (*Pr*<0.50) should generally be maintained on monotherapy or dual bronchodilation unless criteria for ICS use are met. This framework enables systematic audit of treatment adequacy and pharmacological appropriateness. ICS, inhaler corticosteroids; LABA, long-acting beta-agonists; LAMA, long-acting muscarinic antagonist; EOS, blood eosinophil count (cells/μL).

| Seleida Control Status | Expected Treatment Strategy (GOLD-aligned) | Audit Objective |
| --- | --- | --- |
| *Pr*≥0.50 | Escalation to LABA+LAMA or LABA+LAMA+ICS based on eosinophil count | Detect undertreatment, missing ICS indication or suboptimal regimens |
| *Pr*<0.50 | Monotherapy or dual bronchodilation (no ICS unless EOS ≥ 300 + E phenotype or A1/B1 + EOS ≥ 100 depending on the clinical profile) | Identify overtreatment or unnecessary ICS exposure |

Patients flagged as poorly controlled and not receiving triple or dual therapy represent a mismatch requiring clinical review and potentially medication intensification. Conversely, patients with low *Pr* and ICS use without exacerbations or eosinophilia suggest overtreatment, with implications for de-escalation and reduction of systemic risk.

This two-dimensional overlay (*Pr* vs. actual treatment) provides a powerful, real-time quality indicator of adherence to personalized treatment targets at the population level.

#### **Step 2. Infection Risk Surveillance**

In addition to symptom control, Seleida identifies phenotypes associated with chronic antibiotic use (≥5 prescriptions/year). This variable, combined with an E phenotype and high probability of poor control (*Pr*≥0.95), delineates a subgroup at high risk of chronic bronchial colonization—particularly by *Pseudomonas aeruginosa*, which carries important therapeutic and prognostic implications.

The algorithm flags patients meeting the following criteria:

- Seleida phenotype: E (L-Ps) or E (H-Ps).
- ≥5 antibiotics prescribed in the past year.
- *Pr*≥0.95 (indicating high probability of exacerbator status).

These patients are candidates for:

- Order sputum culture (even in the absence of exacerbation).
- Refer to Pulmonology if *Pseudomonas aeruginosa* positive.
- Pharmacological adjustment, potentially including inhaled antibiotic strategies under hospital-based specialist supervision.

#### **Step 3. Integration into Health Systems**

Seleida outputs can be embedded in dashboards to monitor:

- Percentage of patients undertreated vs. overtaken (*Pr* vs. treatment mismatch).
- Monthly high risk alerts.
- Non-adherent prescribing patterns by clinician, center or region.

This approach supports antibiotic stewardship, proactive infection surveillance, and adherence to evidence-based prescribing—all from passive EHR data extraction.

### **Conclusion**

At the institutional level, Seleida operationalizes precision prevention, guideline adherence, and rational prescribing at scale, using only routinely available data.

**S7. FHIR Validation and Interoperability Compliance**

To ensure full compliance with international interoperability standards, the digital application for automated COPD phenotyping was implemented entirely according to the HL7^®^ FHIR^®^ R5 specification. Each Seleida output was encoded as a formal FHIR resource using standardized structures, terminology bindings, and value sets aligned with the FHIR core implementation guide (hl7.fhir.r5.core#5.0.0). The generated resource types included: Patient, RiskAssessment, DetectedIssue, Condition, Provenance, and a structured Bundle of type *collection.*

These resources were generated through automated simulation of valid (*a, b*) input combinations—representing clinically plausible annual SABA/SAMA and antibiotic dispensations—rather than from real patient data. This approach enabled comprehensive technical interoperability testing under HL7^®^ FHIR^®^ standards, while preserving full anonymity and avoiding ethical concerns.

The final output was serialized in JSON format and subjected to comprehensive validation using the official HL7^®^ FHIR^®^ validator engine (v6.5.28), accessible at <https://validator.fhir.org>. A total of 440 individual FHIR resources were included in the validation process. The validator confirmed complete structural, syntactic, and semantic integrity of the bundle: all required fields were properly defined, all inter-resource references (e.g., between RiskAssessment, DetectedIssue, and Condition) were resolvable, and no fatal or error-level messages were reported.

Each resource type served a specific role in reflecting the clinical logic of the Seleida model:

- **RiskAssessment** encoded the predicted probability of poor COPD control and included an interpretable rationale string (e.g., “High rescue and high antibiotic use”). It also specified the computational method (logistic-regression) and linked to the relevant patient and condition entities.
- **DetectedIssue** captured the corresponding clinical recommendation or pharmacologic action derived from the predicted phenotype. Coding fields were aligned with HL7^®^ value set guidance where applicable, and custom extensions were introduced only when no core code existed (e.g., for inferred ICS indication or high rescue use). These were properly documented and declared with appropriate binding strengths.
- **Condition** provided the formal disease context (COPD), coded using SNOMED CT terminology.
- **Provenance** documented the automated generation process, identifying Seleida as the computational agent and specifying that no manual input was involved in resource creation.
- **Patient** was included for structural completeness, although populated with de-identified placeholders given the absence of real data.

The bundle was designed for compatibility with clinical decision support systems and federated EHR environments. Coding elements (system, code, display) followed SNOMED CT, LOINC, and HL7^®^-recommended value sets. All extensions were within permitted scope, ensuring formal compliance.

This validation confirms syntactic and semantic compliance with FHIR and standardized terminologies, supporting safe, interoperable, and scalable integration of the Seleida COPD phenotyping engine into certified digital health infrastructures (Figure S7-1). The architecture ensures semantic traceability, longitudinal linkage of clinical records, and scalable deployment across diverse care settings. The use of simulated data guarantees methodological transparency while demonstrating technical robustness and readiness for real-world implementation under HL7^®^ FHIR^®^ R5.


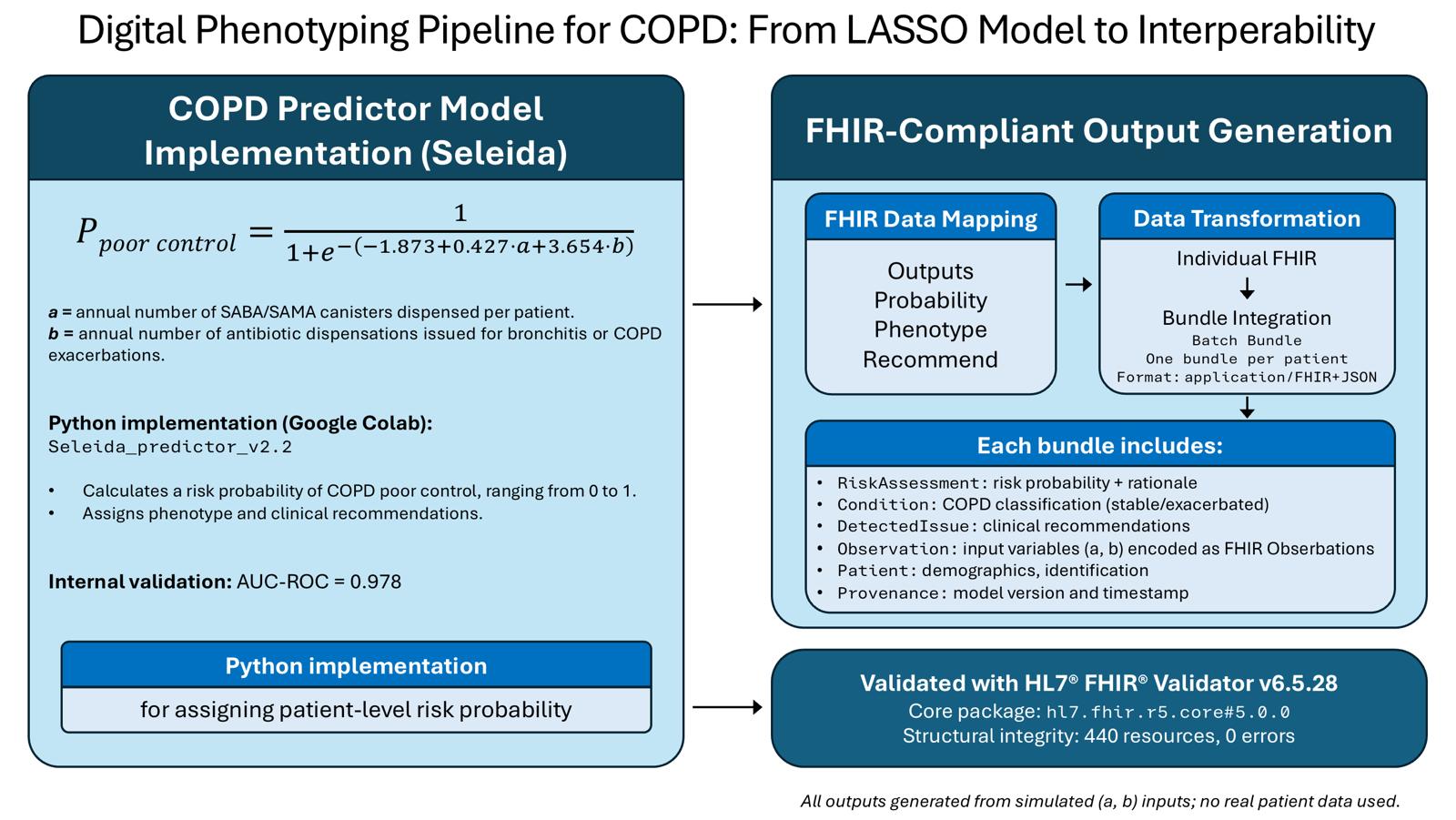


Figure S7-1**.** Simplified flowchart illustrating the deployment architecture of the Seleida COPD predictor. The system integrates a LASSO-regularized logistic regression model (implemented in Python via Google Colab™) with the generation of HL7® FHIR®-structured outputs and export to a FAIR-compliant interface. Each simulated (*a, b*) input pair produces a patient-level FHIR Batch Bundle in JSON format, including: RiskAssessment (predicted probability and rationale), Condition (COPD classification), DetectedIssue (recommendations), Observation (input variables), Patient (demographics and identification), and Provenance (model version and timestamp). Validation with the official HL7® FHIR® Validator (v6.5.28), using the core package hl7.fhir.r5.core#5.0.0, confirmed complete syntactic and semantic integrity across all 440 resources. This architecture ensures full traceability, reproducibility, and certified interoperability for clinical decision support and data exchange.
