# Supplementary figures and images for: Automated Chronic Obstructive Pulmonary Disease Phenotyping and Control Assessment in Primary Care: Retrospective Multicenter Study Using the Seleida Model
Source: JMIR Med Inform. 2025 Oct 13;13:e74932. doi: 10.2196/74932 (PMC12517459; doi:10.2196/74932)

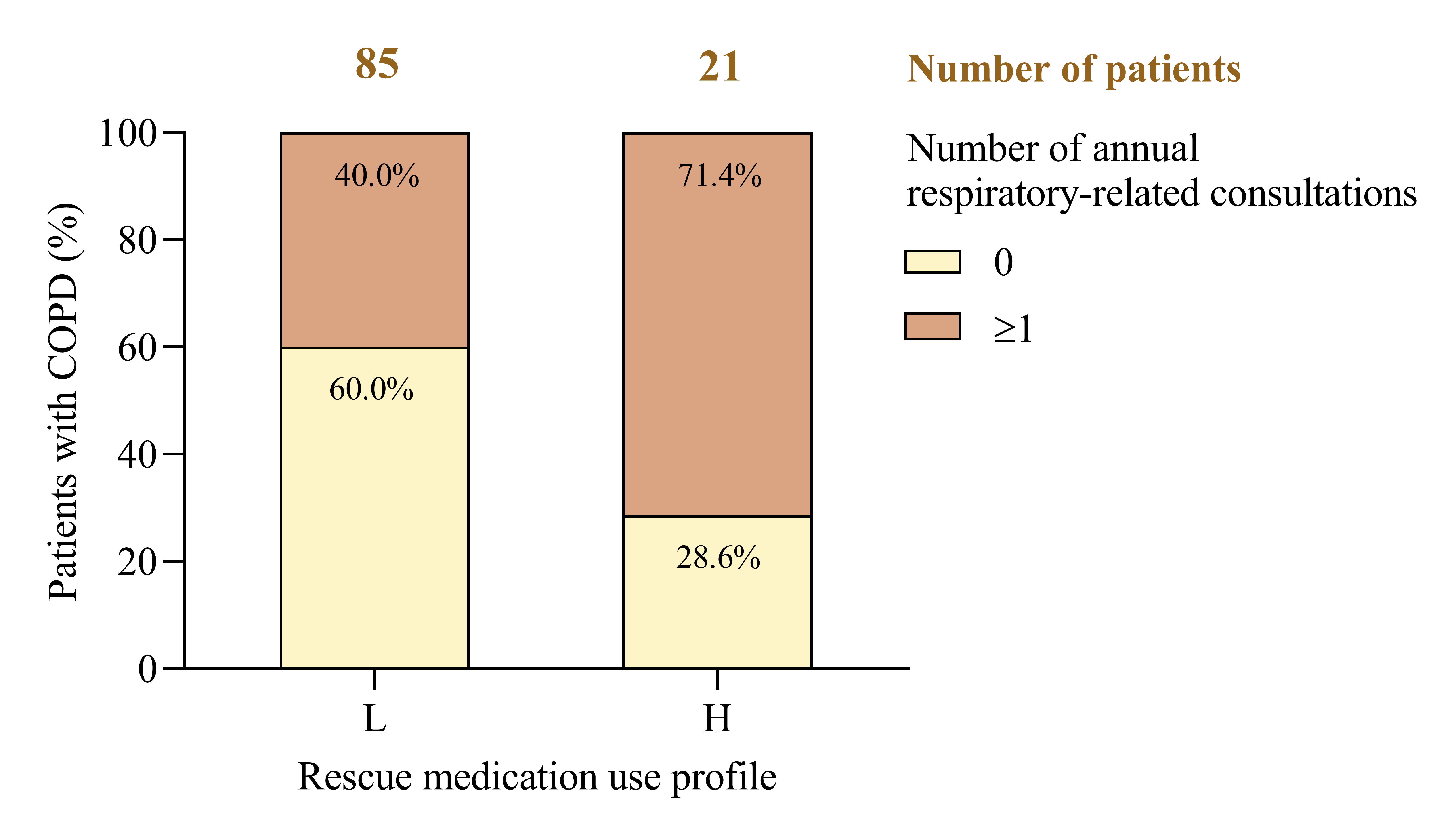

Supplement: Multimedia Appendix 2 [file medinform-v13-e74932-s002.png]

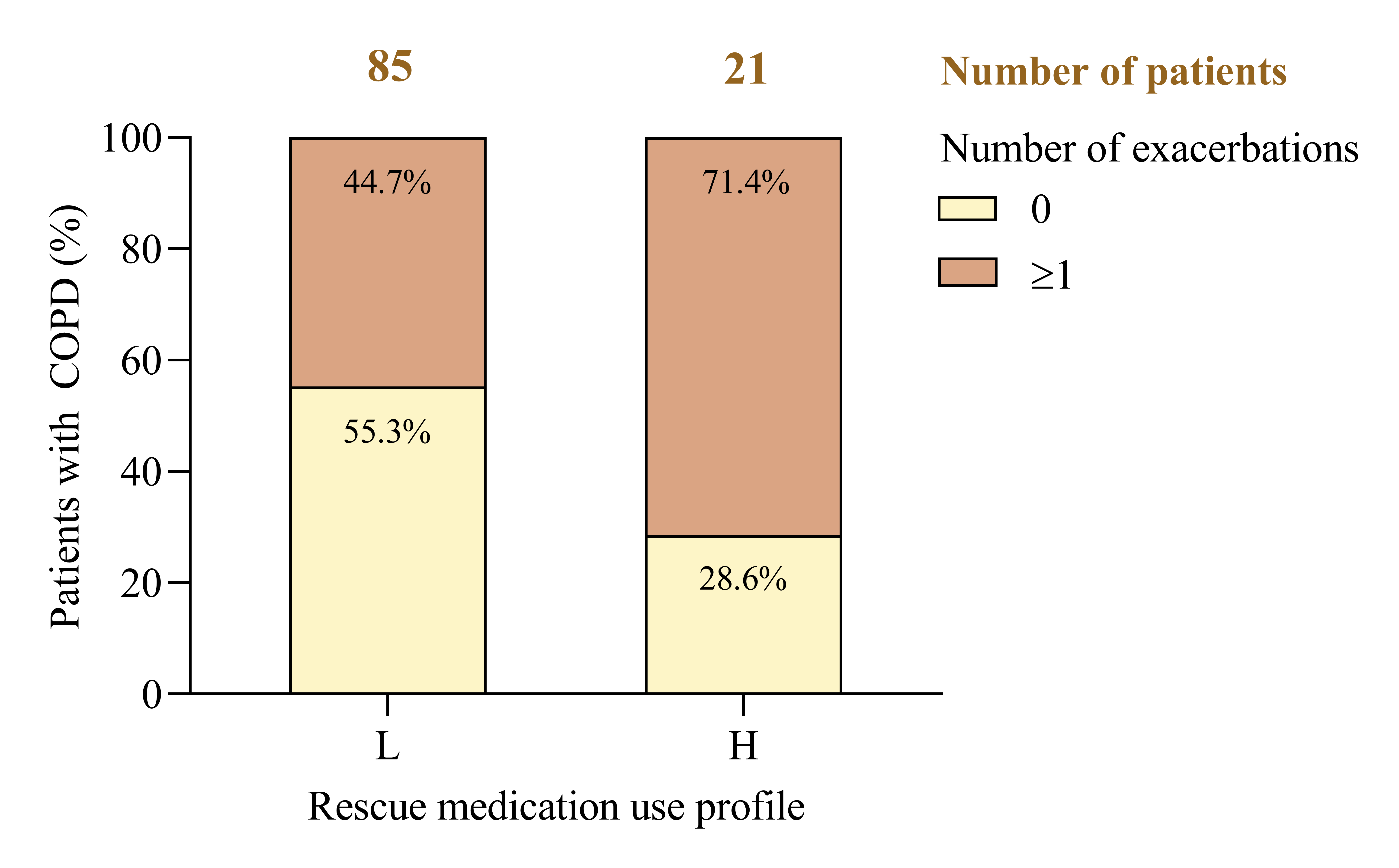

Supplement: Multimedia Appendix 3 [file medinform-v13-e74932-s003.png]
